# Skin‐Integrated Electrogenetic Regulation of Vasculature for Accelerated Wound Healing

**DOI:** 10.1002/advs.202412257

**Published:** 2025-01-10

**Authors:** Preetam Guha Ray, Ragavi Rajasekaran, Bitan Pratihar, Sirshendu De, Santanu Dhara, Martin Fussenegger

**Affiliations:** ^1^ ETH Zurich Department of Biosystems Science and Engineering Klingelbergstrasse 48 Basel CH‐4056 Switzerland; ^2^ Biomaterials and Tissue Engineering Laboratory School of Medical Science and Technology (SMST) Indian Institute of Technology Kharagpur Kharagpur 721302 India; ^3^ Department of Chemical Engineering Indian Institute of Technology Kharagpur Kharagpur 721302 India; ^4^ Faculty of Science University of Basel Klingelbergstrasse 48 Basel CH‐4056 Switzerland

**Keywords:** electrogenetics, gene switch, genetic engineering, synthetic biology, wound healing

## Abstract

Neo‐vascularization plays a key role in achieving long‐term viability of engineered cells contained in medical implants used in precision medicine. Moreover, strategies to promote neo‐vascularization around medical implants may also be useful to promote the healing of deep wounds. In this context, a biocompatible, electroconductive borophene–poly(ε‐caprolactone) (PCL) 3D platform is developed, which is called VOLT, to support designer cells engineered with a direct‐current (DC) voltage‐controlled gene circuit that drives secretion of vascular endothelial growth factor A (VEGFA). The VOLT platform consists of a 3D‐printed borophene‐PCL honeycomb‐shaped matrix decorated with borophene‐PCL nanofibers by electrospinning. The honeycomb structure provides mechanical stability, while the nanofibers facilitate the adhesion, migration, and proliferation of the engineered cells. The cells incorporate a DC‐powered reactive oxygen species (ROS)‐sensing gene circuit wired to an engineered synthetic promoter that triggers secretion of VEGFA to promote vascularization in the adjacent extracellular matrix. Cells engineered with this gene circuit and enclosed in the VOLT matrix, termed the VOLT_VEGFA_ system, can be simply triggered using off‐the‐shelf AA batteries, utilizing the established ability of a brief DC bias to generate non‐cytotoxic levels of ROS. For proof‐of‐concept, a subcutaneous wound‐healing model in rats is chosen. Electrostimulation of a VOLT_VEGFA_ implant (5 V, 20 s per day) induced secretion of VEGFA, and significantly accelerated neovascularization and granulation tissue formation, resulting in faster wound closure compared with non‐stimulated controls. Complete re‐epithelialization and dermal regeneration are observed within 15 days of application.

## Graphical abstract

Herein we introduce the VOLT platform, a three‐dimensional electroconductive borophene‐polycaprolactone matrix supporting engineered cells with a DC‐controlled gene circuit for VEGFA secretion. The system, activated by low DC bias, promotes neovascularization and accelerates wound healing. Tested in a rat wound model, the VOLT_VEGFA_ system demonstrated rapid neovascularization, tissue regeneration and complete wound healing within 15 days, showcasing significant biomedical potential.

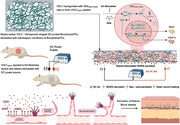

## 1 Introduction

New approaches to treat chronic diseases have emerged in recent years as a result of advances in synthetic biology,^[^ ^1^ ^]^ regenerative medicine,^[^ ^2^, ^3^ ^]^ and T‐cell engineering.^[^ ^4^ ^]^ Programmable open‐loop^[^ ^5^ ^]^ or closed‐loop^[^ ^6^ ^]^ gene circuits enable on‐demand, reversible secretion of therapeutics using a chemical^[^ ^7^, ^8^ ^]^ or physical inducer.^[^ ^6^ ^]^ However, the practical application of live cell implants is still limited by a number of issues, including the need to ensure that implanted engineered cells receive adequate nutrition.^[^ ^9^, ^10^ ^]^ For example, physical damage to the tissue during the insertion of a biomedical implant can impair and delay vascularization.^[^ ^11^ ^]^ In this context, we took a note of the fact that vascular endothelial growth factor A (VEGFA) originating from both tumors and host mesenchymal tissues plays a pivotal role in driving tumor growth by fostering angiogenesis, primarily via activation of the VEGFR‐2 receptor.^[^ ^12^ ^]^ A clinical study showed that percutaneous arterial gene transfer of a plasmid DNA encoding for the 165‐amino‐acid isoform of human VEGF (phVEGF_165_) led to an increase of collateral blood vessels after 4 weeks in a patient with an ischemic limb.^[^ ^13^ ^]^ However, uncontrolled VEGFA expression may lead to edema and tumor formation.^[^ ^13^, ^14^ ^]^ Here, we aimed to develop a voltage‐controlled and reactive oxygen species (ROS)‐responsive gene circuit that can trigger on‐demand secretion of VEGFA, which would then enhance vascularization at the periphery of the bioelectronic device. Furthermore, the designed system also utilizes ROS, which would otherwise have adverse effects on blood vessels,^[^ ^11^ ^]^ to trigger release of VEGFA, thereby promoting the proliferation of blood vessels in the adjacent extracellular matrix.

In humans, ROS originate from the electron‐transfer system in mitochondria and peroxisomes as a part of the natural respiratory process. A ROS‐based transgene expression system using direct current (DC) electrical stimulation of engineered bacterial cells grown on an electrode surface has been reported.^[^ ^15^ ^]^ Further, our group has utilized ROS‐based release of the sequestered nuclear factor erythroid 2–related factor 2 (NRF2) from the NRF2‐Kelch‐like ECH‐associated protein 1 (KEAP1) trimeric complex to design a DC‐actuated regulation technology (DART) device to drive insulin release, and we showed that this system could reverse hyperglycemia in type‐1‐diabetic mice.^[^ ^16^ ^]^ In particular, DC‐powered induction via the generation of non‐toxic levels of ROS can be achieved in just seconds.^[^ ^16^ ^]^ Moreover, the use of off‐the‐shelf batteries makes it robust and convenient for practical application. Here, we used the electro‐genetically active, NRF2/KEAP1/antioxidant response element (ARE)‐based DART system to engineer a gene circuit that regulates electrically driven ROS‐induced secretion of VEGFA.

Another issue is that existing electro‐genetic systems developed to date either for therapeutic or hormone‐sensing applications are built on 2D platforms^[^ ^16^, ^17^, ^18^ ^]^ which can only support low cell densities. It was apparent that to improve long‐term cell viability, proliferation, and migration, as well as stratification into tissue‐like structures, a 3D, structurally stable, and electro‐responsive platform is needed. To design this missing platform for electro‐genetics, we fabricated an electroconductive borophene–poly(ε‐caprolactone) (PCL) matrix decorated with borophene–PCL nanofibers, both to harbor the engineered cells inside a biomimetic micro‐nano environment and to facilitate electro‐stimulation of the cells. In contrast to the original DART system wherein the engineered cells contained in a subcutaneous pocket are stimulated using needles, a 3D fibrous matrix would offer a robust platform for uniform electro‐stimulation while minimizing the potential for aseptic loosening of the engineered cells.

To prepare the matrix, we focused on borophene, which has recently emerged as the lightest 2D nanomaterial with exceptional properties useful in electronics,^[^ ^19^, ^20^ ^]^ optical,^[^ ^21^ ^]^ thermal,^[^ ^22^ ^]^ mechanical,^[^ ^23^ ^]^ and superconducting^[^ ^23^, ^24^ ^]^ applications. This material consists of single‐atom‐thick boron nanosheets with hexagonal voids, composed of two distinct polymorphs, β_12_ and 𝜒_3_. Both polymorphs are predicted to be metallic, with massless Dirac fermion charge carriers, making them attractive for nano‐electronic applications.^[^ ^25^, ^26^, ^27^ ^]^ In contrast to graphene, which possesses structural isotropy, the β_12_ phase is anisotropic, contributing to the high overall carrier density and good mechanical properties of borophene.^[^ ^23^ ^]^ Thus, we aimed to utilize borophene to construct a 3D matrix. In order to scale‐up the synthesis of boron sheets, a liquid‐phase exfoliation (LPE) method was deployed to synthesize multi‐layered borophene using sonication.^[^ ^28^ ^]^ Additionally, in a separate approach, large‐scale synthesis of freestanding borophene, specifically incorporating both χ3 and β_12_ phases–critical for electronic applications–was achieved using a modified LPE method.^[^ ^23^ ^]^ It is already clear that adaption of 2D material to form a 3D matrix has many potential applications in the biomedical sector.^[^ ^29^, ^30^, ^31^, ^32^, ^33^ ^]^ For example, borophene has been blended with Ecoflex membrane to prepare an electroconductive bridging material used to treat acute wounds in rats by means of electrostimulation.^[^ ^34^ ^]^ More recently borophene was molded in a PVA hydrogel with zinc oxide (ZnO) to prepare UV‐cured 3D‐printed mats of borophene‐ZnO for antibacterial and controlled release application.^[^ ^35^ ^]^ Previously, counterparts of borophene like graphene and carbon nanotubes were used to construct multifaceted biomimetic 3D‐architectured biomedical implants with variable pore‐size and enhanced mechanical stability to promote long‐term growth and proliferation of mammalian cells for soft and hard tissue engineering applications.^[^ ^29^ ^]^

Here, we constructed a first‐in‐class 3D platform capable of electro‐genetically regulating DART‐mediated VEGFA secretion in a bio‐electronic implant to promote vascularization. Specifically, an electroconductive 3D‐printed borophene honeycomb scaffold decorated with borophene nanofibers, which we call VOLT, was loaded with engineered mammalian cells and stimulated with direct electric current (DC) to induce secretion of VEGFA. The structure of VOLT promotes the proliferation and migration of the engineered cells while the electronic properties of borophene serve to relay the electrostimulation to program the gene circuit via ROS generation. As proof‐of‐concept, we show that application of the VOLT platform loaded with human cells engineered for VEGFA secretion, called VOLT_VEGFA_, to a full‐thickness subcutaneous wound model in rats accelerated neovascularization and wound repair.

## 2 Results

### 2.1 Design and Characterization of VOLT

To construct VOLT, borophene was first prepared by liquid‐phase sonochemical exfoliation of boron in acetone (Figure 2A). High‐resolution transmission electron microscopy (HRTEM) and selected area electron diffraction (SAED) analysis of the prepared material revealed two distinct crystalline phases (β_12_ and 𝜒_3_ phases) (Figure 2B–D). The set of parallel atomic ridges spaced at 0.51 nm corresponds to the β_12_ phase, while the other set of parallel lattice fringes spaced at 0.42 nm is due to the 𝜒_3_ phase (Figure 2B). These phases, characterized by hexagonal voids arranged in a periodic fashion, represent energetically favorable planar configurations of borophene.^[^ ^19^, ^23^ ^]^ The layered architecture of borophene was further confirmed by means of atomic force microscopy (AFM), which revealed 2D sheets with heights ranging from 1.5 to 2.1 nm (Figure S1A, Supporting Information).

**Figure 1:**
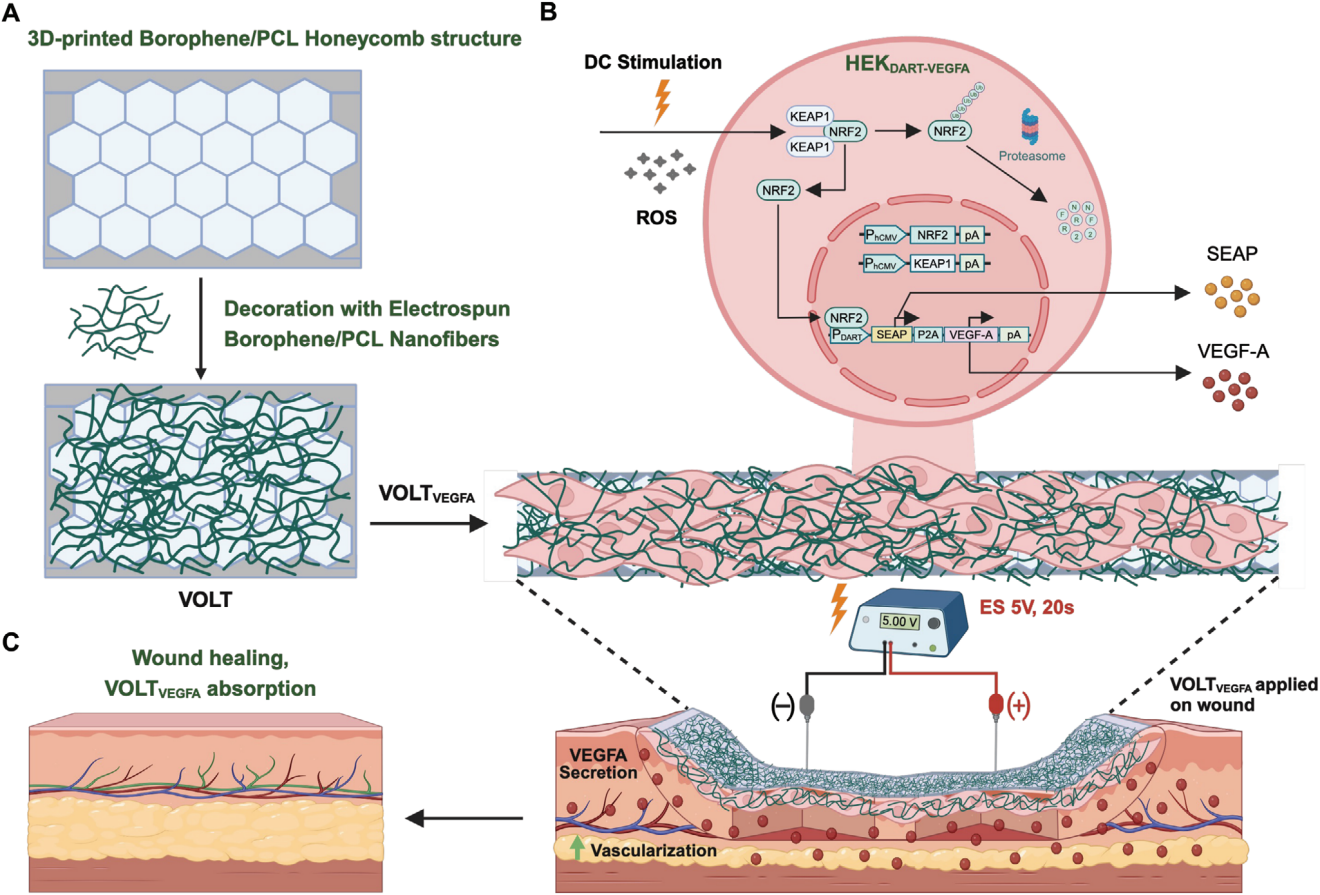
Schematic Illustration of the fabrication and working principle of the VOLT_VEGFA_ system. A) A viscous ink of borophene/PCL was 3D printed into a honeycomb backbone. After overnight drying, the honeycomb backbone was decorated with electrospun borophene/PCL nanofibers to fabricate the VOLT matrices. B) Human embryonic kidney (HEK‐293) cells engineered with DC‐actuated regulation technology (DART) driving secretion of VEGFA were seeded onto the VOLT matrix to form the VOLT_VEGFA_ matrix. C) Electrostimulation of the VOLT_VEGFA_ matrix, implanted on the wound bed, at 5 V DC for 20 s using platinum‐coated needles induced secretion of VEGFA, promoting the formation of new blood vessels in the extracellular matrix leading to accelerated wound healing.

**Figure 2:**
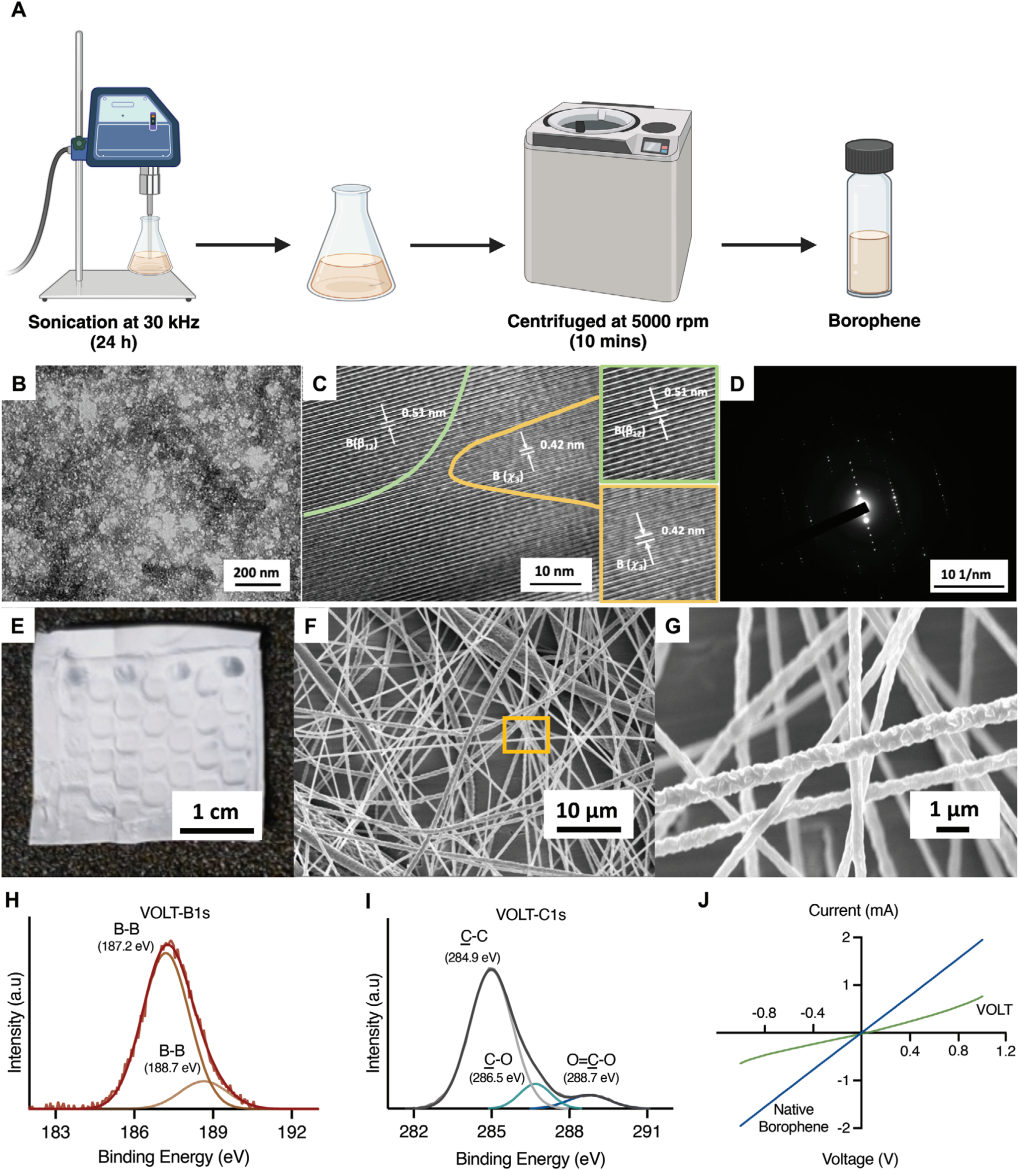
Synthesis, fabrication, and characterization of the VOLT matrix. A) Schematic illustration of the synthesis of borophene by ultrasonication of boron in acetone solution. B,C) HRTEM of borophene confirmed the presence of two polymorphs, β_12_ (*d_spacing_* – 0.51 nm) (border indicated in green) and 𝜒_3_ (*d_spacing_* – 0.42 nm) (border indicated in yellow). D) SAED pattern confirmed the crystallinity of borophene. E) Digital photograph of VOLT. F,G) Field effect scanning electron microscopy micrographs of VOLT confirmed the presence of smooth, well‐defined nanofibers of borophene‐PCL. The highlighted region in F is shown at higher magnification G) to visualize the morphology and integrity of the nanofibers. XPS spectroscopy of the VOLT matrix, showing deconvoluted plots of H) B 1s and I) C 1s in VOLT. J) *I–V* characterization confirmed the electroactive properties of the VOLT matrix, attributable to the presence of borophene.

Next, a honeycomb‐shaped matrix of hexagonal studs was constructed by 3D printing using a borophene (1 mg mL^−1^): PCL (20 wt.%) mixture in chloroform (Movie S1, Figure S1B–D, Supporting Information). The honeycomb borophene structure was decorated with nanofibers in an electrospinning apparatus, using a less viscous borophene (1 mg mL^−1^): PCL (10 wt.%) mixture (**Figure** **1**). The resulting borophene honeycomb structure decorated with nanofibers is the VOLT platform (**Figure** **2**E). Field emission scanning electron microscopy (FESEM) revealed the randomly oriented borophene‐PCL nanofibrous architecture of the VOLT matrix (Figure 2F,G). Analysis of fiber diameter performed using Fiji and GraphPad Prism showed a narrow Gaussian distribution in the range of 200 to 400 nm, which is suitable for proliferation and migration of mammalian cells (Figure S2A, Supporting Information). The prepared VOLT matrix was dissolved in acetone and HRTEM characterization of the dissolved VOLT matrix confirmed the presence of both β_12_ and 𝜒_3_ phases (Figure S2B,C, Supporting Information). This affirmed integrity of the crystalline phases of borophene inside the VOLT matrix. We also characterized and validated the VOLT matrix using X‐ray photoelectron spectroscopy (XPS). The deconvoluted spectrum of core‐level B1s in VOLT exhibited binding energy subpeaks at 187.2 and 188.7 eV (Figure 2H), consistent with B‐B bonding within the borophene phases; in bulk boron, the B 1s peak is typically observed at 189–190 eV.^[^ ^19^ ^]^ The PCL backbone of the matrix showed a C1s binding energy peak at 284.9 eV (Figure 2I). Further, the C1s core‐level spectrum of PCL backbone in VOLT was deconvoluted into three subpeaks at 284.9, 286.5, and 288.7 eV, corresponding to C─C, C─O, and O═C─O groups, respectively (Figure 2I). Similarly, the C1s spectrum of PCL alone showed subpeaks at 284.7 eV (C─C), 286.3 eV (C─O), and 288.5 eV (O═C─O) after deconvolution (Figure S3, Supporting Information). This confirmed the successful incorporation of borophene into the VOLT matrix. I/V characterization of borophene revealed superior electrical conductivity of the 2D material, which can be attributed to the presence of two distinct polymorphs, the β_12_ and 𝜒_3_ phases, in the borophene lattice (Figure 2J). The VOLT matrix showed an average conductivity of 36.25 ± 2.41 mS cm^−1^ in four‐probe resistance measurements of *n* = 5 samples. The presence of borophene in the VOLT matrix imparts it with semiconductor characteristics, enabling it to stimulate the engineered cells.

During incubation in phosphate‐buffered saline (PBS), VOLT exhibited nearly 60% swelling within 3 h, reaching 70% after 10 h. Standard PCL nanofibers (PCL‐NF) showed 23% swelling after 3 h (Figure S4A, Supporting Information). Both VOLT and PCL‐NF reached equilibrium at similar time points, at ≈48 h, when VOLT exhibited swelling of up to 75% due to the presence of borophene, whereas the highly hydrophobic PCL‐NF counterpart showed only 41% swelling. Similarly, VOLT showed faster protein adsorption than PCL‐NF (Figure S4B, Supporting Information), presumably due to the ability of boron to form stable complexes with hydroxyl and amine groups. In this context, it was recently shown that accurate positioning of boron within intricate biomolecular structures enables site‐specific Lewis acid‐base pairing (LABP) of proteins through dative bonding.^[^ ^36^ ^]^ This is consistent with the idea that the presence of borophene facilitates protein adsorption from the cell culture medium, thereby enhancing cell adhesion and growth on the surface of VOLT scaffold. A consistent rise in protein adsorption occurred during incubation, due to the Vroman effect.^[^ ^37^ ^]^ Both VOLT and PCL–NF were slowly degraded in vitro in PBS (pH 7.4) containing 0.5 mg mL^−1^ lysozyme (Figure S4C, Supporting Information).

There was a significant difference in the tensile properties of the VOLT and PCL‐NF matrices (Figure S4D–F, Supporting Information). VOLT exhibited high mechanical stability, with a Young's modulus of 23.6 ± 2.5 MPa and a tensile strength of 11.08 ± 1.8 MPa. In contrast, PCL‐NF had a Young's modulus of 10.34 ± 1.2 MPa and a tensile strength of 5.2 ± 0.9 MPa. The difference is considered to be due to the presence of the β_12_ phase in borophene, which imparts structural isotropy, contributing to the superior mechanical properties of the VOLT matrix.

### 2.2 Cytocompatibility of VOLT Matrix

**Figure 3:**
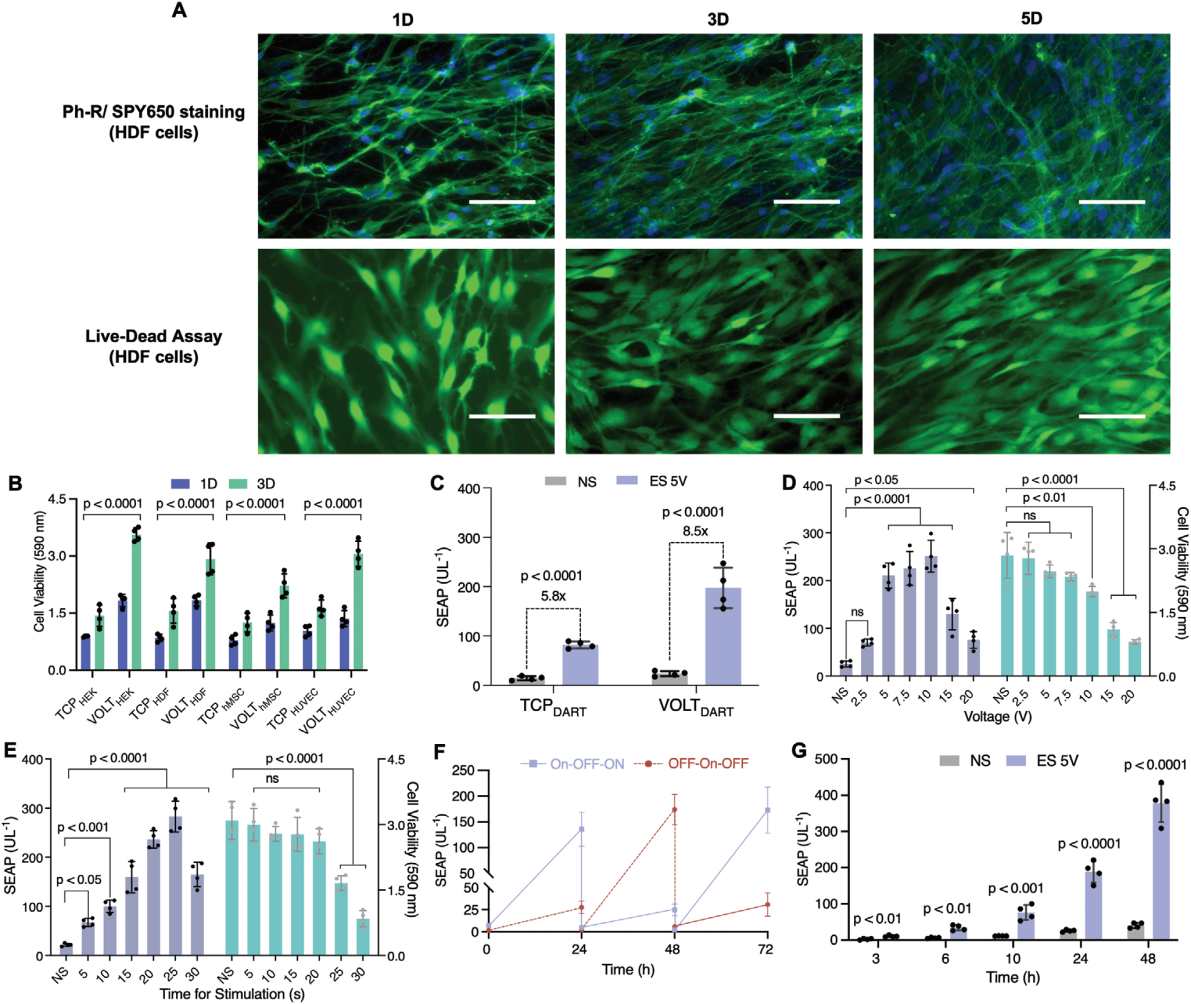
Cyto‐compatibility of the VOLT matrix and activation of the VOLT_DART_ system. A) Proliferation of human dermal fibroblasts (HDF) cultured with the VOLT matrix for 1 (1D), 3 (3D), and 5 (5D) days, was demonstrated by staining the cytoskeleton and nucleus with phalloidin‐rhodamine (green) and SPY‐650 (red), respectively. Cyto‐compatibility of live HDF cells seeded on the VOLT matrix was further validated using a live‐dead assay. Green fluorescence from Calcein AM (live cells) increased over time, indicating cell proliferation, while red fluorescence from EthD‐1 (dead cells) was absent throughout. (Scale bar: 20 µm) B) Metabolic activity of HEK‐293, HDFs, hMSCs, and HUVECs on the VOLT matrix was also confirmed using resazurin assay and compared with the results on a tissue culture plate (TCP) after 1 (1D) and 3 (3D) days of incubation. C) Comparative analysis of the activation of HEK_DART_ cells seeded on top of the VOLT matrix (VOLT_DART_) and on a tissue culture plate (TCP_DART_) under stimulated (ES‐5V for 20s) and non‐stimulated (NS) conditions. D) The VOLT_DART_ system was activated at various voltages for 20 s to study its effect on SEAP production and cell viability. E) Based on the cell viability and SEAP production data, we subsequently employed 5 V for varying time periods (5*–*30s). F) VOLT_DART_ system showed complete reversibility through multiple ON‐to‐OFF and OFF‐to‐ON cycles. G) We profiled the activation kinetics of the VOLT_DART_ system for 48 h. Micrographs were selected from four biologically independent samples. For C), and G), the statistical significance of differences (*p‐*value) was calculated using a two‐tailed, unpaired Student's *t*‐test and for B), D), and E), the *p‐*value was calculated using one‐way ANOVA with Dunnett's multiple comparisons tests. All data are presented as mean ± SD of *n* = 4 biologically independent samples.

To evaluate the cytocompatibility of VOLT, we first exposed 4 different adherent human cells to two different concentrations of native borophene solution. Resazurin assay revealed no cytotoxic effects (Figure S5A, Supporting Information). HEK‐293 cells were also exposed to higher concentrations, and no cytotoxicity was observed (Figure S5B, Supporting Information). In addition, we tested HDF (human dermal fibroblasts), hMSCs (human mesenchymal stem cells), HUVECs (human umbilical vein endothelial cells), and HEK‐293 cells in fabricated VOLT scaffolds as compared to tissue culture plates. The metabolic activity of the cells in the 3D matrix increased with increasing incubation time, clearly demonstrating enhanced proliferation in the 3D matrix over the culture plate (**Figure** **3**B). The proliferation and migration of proliferating HDF were further analyzed using phalloidin‐rhodamine and SPY‐650 staining of the cytoskeleton and nucleus, respectively. Fluorescence micrographs showed that the cells adhered to the nanofibers of the VOLT matrix through their filopodial extensions after 24 h and maintained their elongated spindle shapes with distinct cytoplasmic protrusions (Figure 3A). The HDF cells were able to establish cell‐to‐cell contact without any marked change in their shape or size. By the third day, HDF cells were observed to have migrated throughout the 3D scaffold and formed a sheet‐like structure by the end of 5th day, indicating robust proliferation of the cells and excellent cytocompatibility of the VOLT matrix (Figure 3A). A Z‐stack analysis of fluorescent micrographs confirmed that the HDF cells were able to infiltrate into the VOLT scaffold (Figure S6, Supporting Information). The red fluorescence derived from rhodamine‐phalloidin staining of the cytoplasm observed in the sideways cross‐sectional view further validated the presence of the cells in different layers of the scaffold (Figure S6, Supporting Information). Live dead assay (LD assay) also confirmed negligible cytotoxicity of the VOLT matrix, and the intensity of green fluorescence from Calcein AM, representing live cells, increased progressively with increasing incubation time (Figure 3A). Red fluorescence of the EthD‐1 marker (representing dead cells) was not detectable at any time point (Figure 3A).

### 2.3 Activation of DC‐actuated Regulation Technology Using VOLT

Having established the electroconductive character and cyto‐compatibility of the VOLT matrix, we next deployed an electrogenetically inducible, ROS‐mediated gene circuit powered by direct current (DC)‐actuated regulation technology (DART), developed previously in our group.^[^ ^16^ ^]^ We tested this system with HEK‐293 cells based on their suitability for gene modification and their ability to secrete the expressed product of the gene of interest into the extracellular matrix. The HEK‐293 cells seeded on the VOLT matrix were co‐transfected for constitutive expression of *KEAP1* (pJH1004, P_hCMV_‐*KEAP1*‐pA), and *NRF2* (pJH1003, P_hCMV_‐*NRF2*‐pA) vectors linked to a DART‐based reporter construct pJH1005 (P_DART_‐SEAP‐pA; P_DART_, O_ARE_‐P_hCMVmin_). This expression system, termed VOLT_DART_ is designed so that *NRF2* is released only in presence of ROS, enabling it to bind to the operator (O_ARE_) to initiate downstream regulation of the gene encoding SEAP (human placental secreted alkaline phosphatase) as a marker that can be measured in the supernatant. In a control experiment, we confirmed that *NRF2* can activate the *ARE* operator (Figure S7, Supporting Information). However, inducer‐mediated regulation of pJH1005 is only possible in the presence of all three vector components (pJH1003, pJH1004, and pJH1005) (Figure S7, Supporting Information). To validate the VOLT_DART_ system, we performed a comparative analysis with DART‐expressing cells seeded on a tissue culture plate (TCP) and stimulated with a DC bias (5 V, 20 s).^[^ ^16^ ^]^ The VOLT_DART_ system showed superior activation dynamics when stimulated with DC, with more than 2‐fold higher induction as compared to the DART cells in culture plates (Figure 3C). This may be due at least in part to increased cell proliferation in the 3D VOLT matrix (Figure 3B).

**Figure 4:**
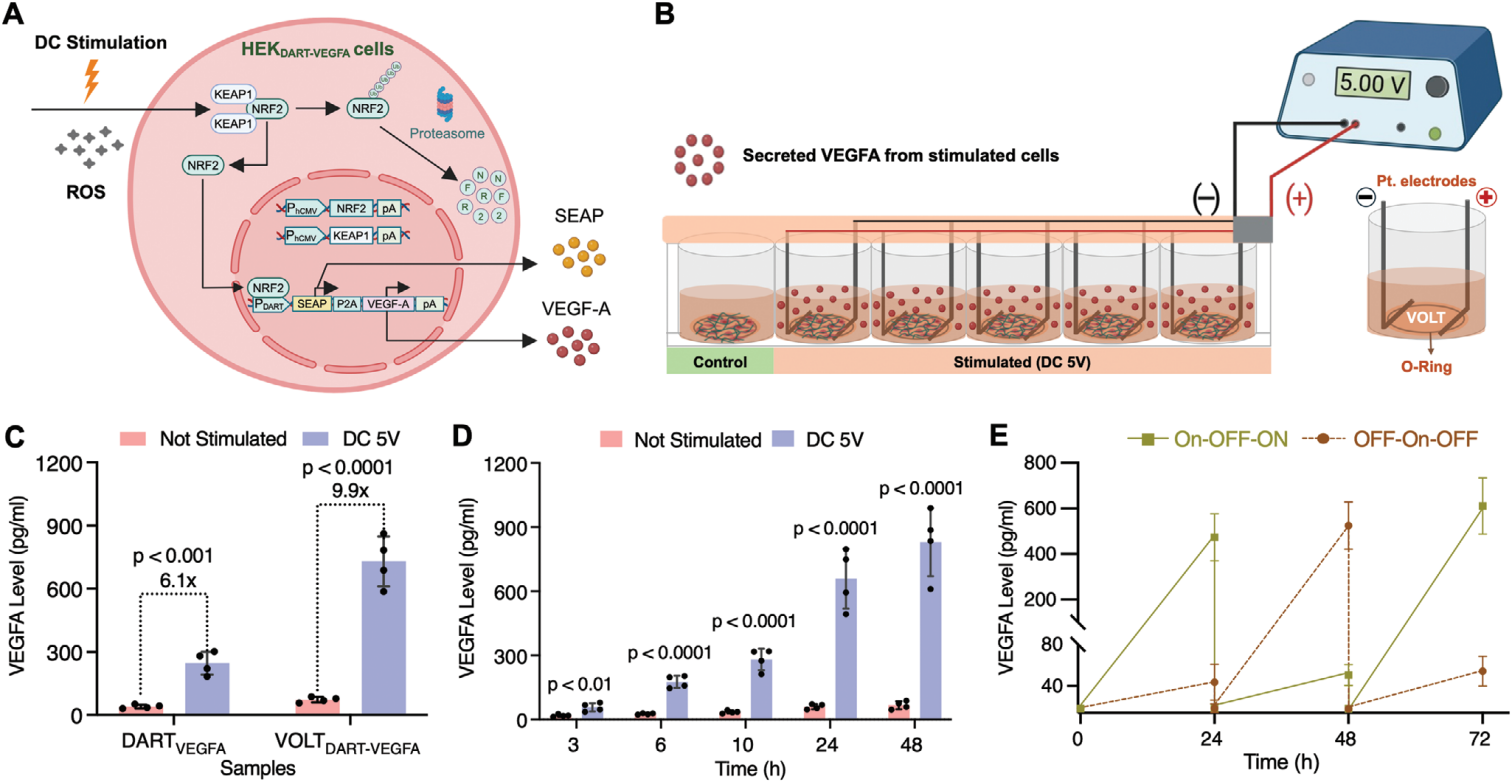
Electrogenetic regulation of VOLT_VEGFA_. A) Schematic illustration of the DART electrogenetic circuit relying on NRF2/KEAP1/ARE to drive secretion of VEGFA from stable monoclonal HEK‐293 cell lines (HEK_DART‐VEGFA_ cells) when stimulated with DC bias. HEK_DART‐VEGFA_ cells were seeded onto the VOLT matrix to fabricate the VOLT_VEGFA_ system. B) Schematic illustration of electrostimulation of the VOLT_VEGFA_ system in a 24‐well plate format. C) Expression of VEGFA in the cell culture supernatant was far higher in the 3D VOLT_VEGFA_ system when compared with HEK_DART‐VEGFA_ cells cultured on 2D tissue culture plates (TCP‐HEK_DART‐VEGFA_). D) The VOLT_VEGFA_ system demonstrates consistent VEGFA secretion kinetics over a period of 10 days (72*–*240 h shown in Figure S10C). E) Reversibility of the VOLT_VEGFA_ system was established during ON‐OFF‐ON or OFF‐ON‐OFF switching. Data are presented as mean ± SD of *n* = 4 biologically independent samples. The statistical significance of differences (*p‐*value) was calculated using a two‐tailed, unpaired Student's *t*‐test.

Next, to optimize the operating parameters, the behavior of the VOLT_DART_ system was characterized at different voltages for varying durations using two L‐shaped parallel platinum electrodes (0.5 mm) separated by 6 mm to generate ROS (**Figure** **4**B). The highest SEAP expression levels were obtained at 5–10 V and dropped significantly at higher voltages (Figure 3D). Therefore, 5 V was chosen for subsequent experiments. The level of SEAP expression also increased with increasing stimulation time, reaching a maximum at 20–25 s (Figure 3E). Furthermore, the VOLT_DART_ system exhibited consistent induction and repression profiles through multiple ON‐to‐OFF and OFF‐to‐ON cycles (Figure 3F). Under the optimized conditions, electro‐stimulated protein production in the culture supernatant was induced within 3 hours, and effective levels of biopharmaceutical expression were achieved after 6–10 h, matching the behavioral profile of professional secretory cells (Figure 3G).

### 2.4 Electro‐genetic Regulation of VOLT_VEGFA_

The VOLT_DART_ system was next applied to drive the secretion of VEGFA. The reporter vector was engineered to add the *VEGFA* gene, in addition to the SEAP expression system (P_DART_‐*SEAP*‐P2A‐*VEGFA*‐pA; pPGR30). We co‐transfected pPGR30 transiently with pJH1003 (P_hCMV_‐*NRF2*‐pA) and pJH1004 (P_hCMV_‐*KEAP1*‐pA) following a procedure similar to that used for the VOLT_DART_ system. The resulting system was termed VOLT_DART‐VEGFA_ (Figure 4A). The VOLT_DART‐VEGFA_ system was electro‐stimulated at 5 V for 20 s, then incubated for 24 h, and VEGFA expression in the supernatant was analyzed by means of ELISA (Figure 4B). We observed significantly increased levels of VEGFA in the VOLT_DART‐VEGFA_ system after electrostimulation as compared to its non‐stimulated counterpart (Figure 4C). Further, the 3D VOLT_DART‐VEGFA_ system showed threefold greater VEGFA expression as compared to the 2D system in a standard 24‐well plate. We also engineered hMSCs seeded on the VOLT matrix with the same expression cassettes to study the expression of VEGFA (designated as VOLT_DART‐VEGFA‐hMSC_) and SEAP (hereafter termed as VOLT_DART‐hMSC_). Stimulation of the resulting system at 2.5–10 V resulted in stable expression of VEGFA and SEAP in the cell culture medium (Figure S8A,B, Supporting Information). However, since expression of both VEGFA and SEAP was higher in the VOLT_DART‐VEGFA_ and VOLT_DART_ systems, respectively, these were selected for further studies. Since we envisaged long‐term application, we deployed a Sleeping Beauty transposon system to engineer HEK‐293 cells for stable expression of the designed vector system. HEK‐293 cells seeded in a 6‐well plate were co‐transfected with pJH1101 (ITR‐P_hCMV_‐*NRF2*‐pA:P_RPBSA_‐*ECFP*‐P2A‐*PuroR*‐pA‐ITR), pJH1054 (ITR‐P_hCMV_‐*KEAP1*‐P2A‐BlastR‐pA‐ITR) and pPGR35 (ITR‐P_DART4_‐*SEAP*‐P2A‐*VEGFA*:P_hCMV_‐*ZeoR*‐P2A‐*mRuby*‐pA‐ITR). Monoclonal cell lines expressing the complete vector system were selected using puromycin, blasticidin, and zeocin resistance. The monoclonals were electrostimulated at 5 V for 20 s to select the best clone based on SEAP expression (Figure S9A, Supporting Information). The best‐performing clone, no. 8, was also tested for VEGFA and SEAP expression to confirm its activity (Figure S9B,C, Supporting Information). This cell line, termed HEK_DART‐VEGFA_, was confirmed to be monoclonal by FACS analysis.

HEK_DART‐VEGFA_ cells were seeded on the VOLT matrix to prepare the VOLT_VEGFA_ system. We confirmed that, like the VOLT_DART_ system, the VOLT_VEGFA_ system afforded optimal expression of VEGFA in the voltage range of 5–10 V for 20 s (Figure S10A,B, Supporting Information). VEGFA was present in the cell culture medium at 3 h after electro‐stimulation and therapeutically significant levels were achieved by 6 h (Figure 4D). Profiling of the secretion kinetics for 10 days revealed a continuous increase in VEGFA levels, which should be suitable to induce vasculature in a tissue matrix (Figure 4D; Figure S10C, Supporting Information).^[^ ^38^ ^]^ The VOLT_VEGFA_ system also demonstrated continuous growth of the engineered cells over a period of 12 days (Figure S11, Supporting Information). Under in vivo conditions, VEGFA would be cleared from the circulation and would need to be replenished periodically to facilitate complete vascularization. To model this situation, we tested the reversibility of the DC‐induced transcriptional activity of the VOLT_VEGFA_ system. VEGFA levels profiled during ON‐to‐OFF and OFF‐to‐ON induction cycles demonstrated complete reversibility of the system (Figure 4E). Evaluation of the stimulated VOLT_VEGFA_ system (DC 5 V, 20 s) implanted subcutaneously in rats established that elevated expression of VEFGA was maintained for at least 20 days without cell replacement (Figure S12A, Supporting Information). A weak immune response was observed at 5 days after implantation, but there was no significant difference from the control at 10 or 15 days (Figure S12B,C, Supporting Information).

### 2.5 Validation of VOLT_VEGFA_ in a Subcutaneous Wound Model

**Figure 5:**
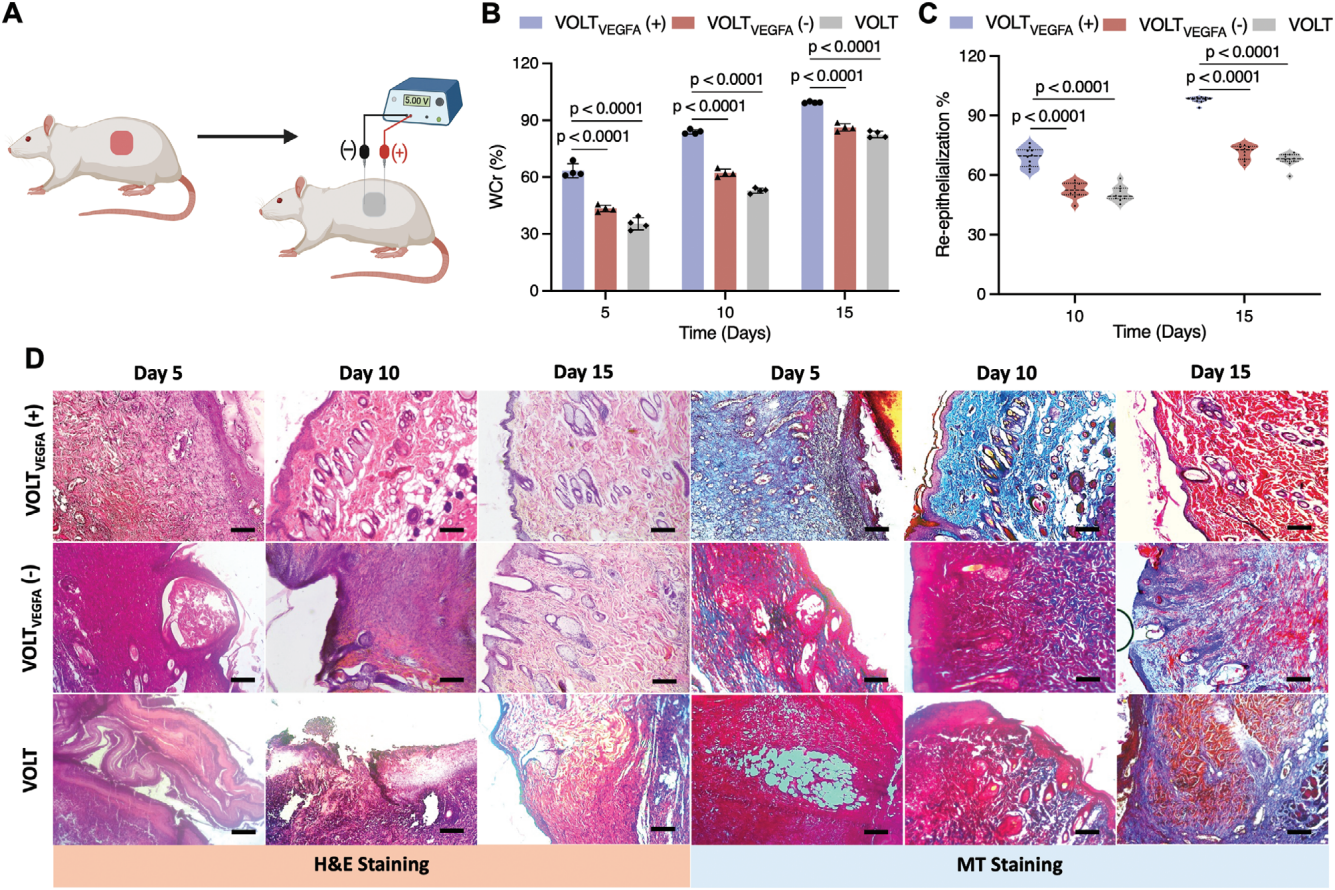
Evaluation of vasculature during full‐thickness subcutaneous wound healing in Wistar rats. A) Schematic illustration depicting electrostimulation of the VOLT_VEGFA_ scaffold with a DC source generator (5 V, 20 s) to treat a subcutaneous full‐thickness wound in Wistar rats. B) The wound closure rate (WCr%) was calculated from digital photographs of the healed wound bed (presented in Figure S13A). C) The % re‐epithelialization was calculated from the histological evaluation of retrieved tissues on days 5, 10, and 15 after H&E staining. D) H&E and MT‐stained micrographs of tissue slices of the healed tissues recovered after 5, 10, and 15 days. (Scale bar: 200 µm). Data are presented as mean ± SD of *n* = 4 biologically independent samples. Micrographs are representative of 4 biologically independent samples. The statistical significance of differences (*p‐*value) was calculated using one‐way ANOVA with Dunnett's multiple comparisons tests.

**Figure 6:**
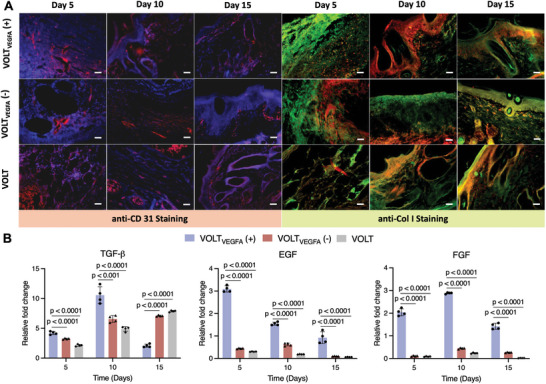
Evaluation of vasculature and collagen deposition during full‐thickness cutaneous wound healing in Wistar rats. A) Immunohistochemical (IHC) staining of healed tissue sections retrieved at days 5, 10, and 15 after implantation was carried out using anti‐CD31 and anti‐COL I antibodies supplemented with DAPI staining (Scale bar: 100 µm). B) The relative fold change in expression of TGF‐β, EGF and FGF genes was evaluated from RT‐PCR of excised tissue samples at different time points (days 5, 10, and 15). Micrographs are representative of 4 biologically independent samples. Data are presented as mean ± SD of *n* = 4 biologically independent samples. The statistical significance of differences (*p‐*value) was calculated using one‐way ANOVA with Dunnett's multiple comparisons tests.

In chronic wound models, damage to primary and auxiliary blood vessels may lead to scab formation on the wound bed, delaying the regeneration process and potentially leading to secondary infections. Here, as a proof‐of‐concept application of the VOLT_VEGFA_ system for electro‐genetic promotion of vascularization and healing, we deployed the system in a subcutaneous wound model in rats. The VOLT_VEGFA_ matrix was fixed on the wound bed, and two platinum‐coated needles were used to electrostimulate the VOLT_VEGFA_ system using a DC source generator (5 V, 20 s) (**Figure** **5**A). Photographs of the wound‐healing process are shown in Figure S13A (Supporting Information) and were used to calculate the wound closure rate. Wound closure was significantly faster in the electro‐stimulated VOLT_VEGFA_ (+) animals as compared to non‐stimulated controls (Figure 5B). In the VOLT_VEGFA_ (+) group, 63% wound closure was attained within 5 days while the corresponding values for the VOLT_VEGFA_ (−) and VOLT groups were only 43% and 35%, respectively. The wound area was reduced by 83% at day 10, and complete wound closure was observed after 15 days in the VOLT_VEGFA_ (+) group (Figure 5B). The matrix bridged the wound bed and supported faster migration of dermal fibroblasts, leading to the formation of granulation tissue (Figure 5D, **H&E‐5 day**). The VOLT_VEGFA_ (−) and VOLT groups achieved only 82% and 80% wound closure, respectively, after 15 days (Figure 5B). The VOLT_VEGFA_ (+) group also showed 68% re‐epithelialization efficiency within 10 days, and complete re‐epithelialization was achieved in 15 days (Figure 5C). The VOLT_VEGFA_ (−) and VOLT groups had significantly lower re‐epithelialization rates of 52% and 50% at 10 days, and 71% and 67% after 15 days, respectively. H&E micrographs also confirmed successful integration of the VOLT_VEGFA_ system with the formation of granulation tissue after 5 days (Figure 5D, **H&E‐5 day**). Anti‐CD 31 staining of the tissue revealed extensive formation of neo blood vessels, seen as red dots, in the region (**Figure** **6**A anti‐CD 31–5 days). Subsequently, enhanced proliferation of dermal fibroblasts resulted in faster collagen deposition in the wound (blue regions of the MT‐stained micrographs; Figure 5D). Immuno‐histochemical staining with anti‐Col I marker also confirmed extensive collagen distribution in the VOLT_VEGFA_‐treated group (Figure 6A).

CD31 serves as a biomarker for circulating angio‐vasculogenic cells, primarily endothelial cells.^[^ ^39^ ^]^ After 10 days, CD31 mapping in VOLT_VEGFA_‐treated samples highlighted re‐alignment of the red dots into round‐shaped blood vessel‐like structures (Figure 6A, **anti‐CD31‐Day 10**). Finally, after 15 days of incubation, the neo‐vessels and the CD31‐positive dots were replaced by continuous red lines, representing mature blood vessels (Figure 6A). In non‐stimulated animals, CD 31 expression was initially very weak, becoming more prominent by the 10th day and intensifying further after 15 days (Figure 6A). These results indicate that electro‐stimulated VEGFA expression induced faster neovascularization and formation of mature blood vessels in this model. The vessel density was evaluated from anti‐CD 31 immuno‐stained micrographs using Image J software. There was a statistically significant increase in blood vessel density (mm^2^) in the VOLT_VEGFA_ (+) group after 5 and 10 days of stimulation as compared to the non‐stimulated counterpart (Figure S13B, Supporting Information). Subsequently, the neo‐blood vessels in the healing region developed into mature blood vessels, resulting in a decrease of the overall vessel density. This further confirms that electro‐stimulated expression of VEGFA facilitates faster neovascularization and formation of mature blood vessels in the surrounding area.

Histochemical staining of tissue extracts from the treated group after 10 days confirmed the development of skin appendages and the alignment of the collagen bundles, consistent with the formation of dermis (Figure 5D, H&E, MT‐10 day). H&E micrographs also showed cellular infiltration in the VOLT_VEGFA_ system and stratification of the cells in the process of re‐epithelialization (Figure 5D, **H&E‐10 day**). In comparison, infiltration of granulation tissue in the non‐stimulated group was only observed after 10 days (Figure 5D, **H&E‐Day 10**).

Furthermore, sparse and unorganized collagen deposition and distribution were observed in the healing matrix of the untreated group, which was in accordance with the micrographs of anti‐Col I antibody staining (Figure 5D, **MT‐10 day**, Figure 6A **anti‐Col I‐Day 10**). Finally, tissue sections from the VOLT_VEGFA_‐treated group on day 15 showed re‐alignment of the collagen bundles into “angel‐curl‐shaped structures” resembling those seen in mature human skin (Figure 5D, **MT‐15 day**).^[^ ^40^ ^]^ Reconstitution of the collagen fibrils in the VOLT_VEGFA_ group was also corroborated by anti‐Col I staining (Figure 6A, **anti‐Col I‐15 day**). The development of mature skin appendages and complete re‐epithelialization along with the formation of mature blood vessels confirmed the effective closure and regeneration of the wound bed (Figure 5D, **H&E‐15 day**, Figure 6A, **anti‐CD31‐Day 15**). In the non‐stimulated group, immature skin appendages and blood vessels with non‐aligned collagen deposits suggested delayed fibroblast migration and granulation tissue formation (Figure 5D, **H&E‐10 day**). Overall, these results indicate that electrostimulation of VOLT_VEGFA_ guided neo‐vascularization in the early stages, thereby promoting fibroblast proliferation in the granulation tissue, leading to faster regeneration of mature dermis.

### 2.6 RT‐PCR Analysis of Gene Expression in Healing Tissues

Biomarker expression in the healing tissue was monitored by RT‐PCR. At 5 and 10 days of treatment, TGF‐β1 gene expression was significantly increased in the VOLT_VEGFA_ (+) group as compared to VOLT_VEGFA_ (−) or VOLT (Figure 6B), then declined to baseline by day 15. EGF gene expression in the VOLT_VEGFA_ (+) group peaked at 5 days and gradually returned to baseline by 15 days (Figure 6B). FGF gene expression was increased at days 5 and 10 in the VOLT_VEGFA_ (+) group but declined by day 15 (Figure 6B).

EGF plays a pivotal role in the epithelialization of healing tissues in a wound bed by supporting cell proliferation and migration to repair injured or damaged skin tissue. TGF‐β1 is essential for the healing process and together with FGF, promotes angiogenesis by modulating the extracellular matrix and stimulating endothelial cell migration for the formation of new blood vessels. FGF stimulates fibroblasts to synthesize ECM components such as collagen and proteoglycans, which provide structural support to the newly forming tissue. Thus, the changes in expression of these genes in the VOLT_VEGFA_ (+) group are consistent with faster re‐epithelialization and angiogenesis in the healing tissues. The micrographs in Figure 6A are also consistent with activation of endothelial cells by VEGFA secreted from the engineered cells, resulting in early neo‐vascularization and formation of new blood vessels.

Collagen synthesis, deposition, and maturation are critical during the wound‐healing process to ensure proper formation of dermis and prevent scar formation. Expression of Col III and Col I was elevated in the VOLT_VEGFA_ (+) group as compared to controls until Day 10 (Figure S14A,B, Supporting Information). By Day 15, expression of Col III and Col I decreased in VOLT_VEGFA_ (+) group. This confirmed maturation of dermis along with the staining micrographs and also assured the prevention of scar formation after completion of the healing process. We also observed elevated expression of pro‐inflammatory cytokines IL‐6, which promotes migration of fibroblasts and keratinocytes, and TNF‐α, which stimulates angiogenesis, in the early stages of wound healing in the VOLT_VEGFA_ (+) group (Figure S14C‐D, Supporting Information). The levels of these pro‐inflammatory cytokines subsequently decreased almost to baseline levels at 15 days.

## 3 Discussion

Ensuring adequate neo‐vascularization is a key challenge in achieving long‐term viability and integration of cell‐based implants and regenerative therapies, as well as being critical for deep wound‐healing. Here, we describe a first‐in‐class device, consisting of an electroconductive scaffold decorated with nanofibers loaded with cells engineered to express VEGFA in response to brief DC stimulation, thereby promoting vascularization in the adjacent extracellular matrix. Engineered‐cell‐based bio‐electronic devices have already been employed not only to control transgene expression to reverse type‐1 diabetes but also to detect hormones in blood samples.^[^ ^6^, ^16^, ^17^, ^18^ ^]^ However, these devices employ a 2D interface, which limits cell migration and proliferation, as well as vascularization, during long‐term application. FDA‐approved encapsulation technologies have already revolutionized the delivery of therapeutic cells,^[^ ^41^ ^]^ but issues such as lack of stability, limited oxygen and nutrient diffusion, immunological reactions, material biocompatibility, batch‐to‐batch variation and inability to integrate into host tissue impede their therapeutic efficacy and have prevented widespread adoption.^[^ ^42^ ^]^ To overcome these limitations, we constructed the 3D VOLT matrix by 3D‐printing a honeycomb‐shaped borophene/PCL matrix and decorating it with borophene/PCL nanofibers by electrospinning. PCL is an FDA‐approved biocompatible biomaterial that not only enhances the cytocompatibility of the matrix but also facilitates the fabrication process. This matrix effectively supported cells engineered with a DC‐actuated transgene expression system, DART, that drives secretion of VEGFA in the extracellular matrix to promote vascularization in the surrounding tissue in response to ROS generated by the applied DC bias. The newly formed blood vessels provide the therapeutic cells with better access to essential nutrients from the host, increasing the longevity of the implant. The 3D spatial arrangement of nano‐microfibers provides a suitable matrix to promote cell adhesion, proliferation, and migration, and the electroactive property of the VOLT matrix allows easy induction of VEGFA from the engineered cells. The DC bias generates low levels of ROS to activate transgene expression, and it was previously confirmed that the resulting ROS generation has no significant impact on cell viability, or on the cell transcriptome.^[^ ^16^ ^]^ The DART device is based on ectopic expression of endogenous KEAP1 and NRF2 trimeric protein complex that redirects ROS detection through synthetic P_DART_ promoters, facilitating the expression of biopharmaceuticals.^[^ ^16^ ^]^ We re‐engineered the operator/promoter to express both VEGFA and a second protein of interest (the marker SEAP in the case of HEK_DART‐VEGFA_ cells). We confirmed that HEK_DART‐VEGFA_ cells seeded on the VOLT matrix (the VOLT_VEGFA_ system) can proliferate efficiently and electro‐genetically secrete VEGFA, thereby promoting vascularization. The system can be reversibly tuned while maintaining a steady secretion dynamic of the therapeutic protein over a long period of time. A significant advantage of the system lies in the fact that it utilizes detection of ROS, which is known to have a negative impact on blood vessels, to express VEGFA that promotes vascularization. The gene circuit was also tested successfully in hMSCs, but in line with our previous findings,^[^ ^16^ ^]^ the HEK_DART‐VEGFA_ cells showed better secretion kinetics and were therefore chosen to build the VOLT_VEGFA_ system.

The biomimetic architecture of VOLT matrix allows stable integration to the host tissue while its mechanical robustness provides long‐term stability in vivo and under cell culture conditions. We deployed the electrogenetic regulation of VEGFA expression from the VOLT_VEGFA_ system in a proof‐of‐concept wound healing experiment in a rat model. Briefly, DC electro‐stimulation accelerated wound closure, which was complete within 15 days, with re‐epithelialization, collagen deposition, and the formation of mature blood vessels. We previously found that only 60–70% wound closure was attained after 21 days in untreated controls,^[^ ^40^ ^]^ whereas complete wound closure was achieved here within 15 days in the VOLT_VEGFA_ (+) group (Figure 5B and Figure S13A, Supporting Information). Furthermore, wounds treated with VOLT_VEGFA_ (+) demonstrated neovascularization within the first 5 days of treatment, presumably due to the secretion of VEGFA, whereas neo‐vessels were only sparsely distributed in untreated wound beds even after 5–10 days.^[^ ^40^ ^]^ Indeed, a rapid increase in blood vessel density was seen in the VOLT_VEGFA_ (+) group in the first 10 days and subsequently decreased due to the maturation of blood vessels. Commercially used FDA‐approved non‐autologous implants such as Matrigel lack a well‐defined structure, which brings into question their stability and integrity in in vivo applications, as well as the long‐term stability in stimulated drug‐testing conditions. Moreover, Matrigel is prepared from ECM derived from Engelbreth‐Holm‐Swarm (EHS) mouse sarcoma cells,^[^ ^43^ ^]^ and consequently is immunogenic in the host,^[^ ^44^ ^]^ increasing the likelihood of fibrosis around the implant. Further, the use of biologically sourced materials as the backbone in Matrigel^[^ ^43^ ^]^ or alginate^[^ ^45^ ^]^ beads leads to batch‐to‐batch variability and a lack of reproducibility in large‐scale manufacturing.

We believe our VOLT technology represents a significant advancement in the state‐of‐the‐art for targeted delivery of engineered cell therapies. We anticipate that this system will effectively promote neo‐vascularization around implants in cell‐based therapies, which should increase their longevity. Our in vivo proof‐of‐concept study supports this view and indeed suggests that the VOLT technology has potential for clinical translation to promote wound‐healing. Importantly, our system can simultaneously express a second gene of interest (the marker SEAP in our study), besides VEGFA, thus providing a range of therapeutic options. The developed VOLT_VEGFA_ system might also have potential for application as a vascular graft. We are currently planning further work to explore these possibilities.

## 4 Experimental Section

### Molecular Cloning and DNA Constructs

Details of the design and construction of the expression vectors are provided in Table S1 (Supporting Information).

### Cell Culture and Transient Transfection. Cell Culture

Human embryonic kidney cells (HEK‐293T, ATCC: CRL‐3216), human dermal fibroblasts (HDFs), human mesenchymal stem cells (hMSCs), and HepG2 cells were cultured in Dulbecco's modified Eagle's medium (DMEM; Life Technologies, Carlsbad, CA, USA) supplemented with 10% (v/v) fetal bovine serum (FBS; Sigma‐Aldrich, Munich, Germany) and 1% (v/v) penicillin/streptomycin solution (PS: Sigma‐Aldrich, Munich, Germany) in a humidified atmosphere (relative humidity (RH) 85%) of 5% CO_2_ in air at 37 °C. Cell density was assessed using brightfield optics and a DirectPipette‐based device (CellDrop, Labgene Scientific SA, Châtel‐Saint‐Denis, Switzerland). Human umbilical vein endothelial cells (HUVECs) were isolated from umbilical cords following collagenase type I treatment,^[^ ^46^ ^]^ in compliance with the Institute's Ethical Committee guidelines (ethics approval number IIT/SRIC/DR/2019, dated February 27, 2019). Post‐isolation, the cells were cultured and maintained in phenol‐red free M200 medium (Thermo Fisher Scientific, India) supplemented with low serum growth supplement (LSGS, Thermo Fisher Scientific, India) and 1% antibiotic solution, under 5% CO_2_ in air at 37 °C. HUVECs cultured between passage numbers 2 and 4 were used in the experiments. For harvesting, cells were treated with trypsin (Himedia, India) for 40 s, followed by centrifugation at 2500 rpm for 3 min, after which the cells were resuspended in the above medium for subsequent experiments. **Transfection**. Transfection of HEK‐293 cells and hMSCs was performed according to a standard protocol in a 24‐well plate format. A transfection mixture containing 500 ng of total plasmid DNA in 200 µL of reduced‐serum Opti‐MEM media (Gibco, USA), and 1.25 µL of polyethyleneimine (PEI; Polysciences Inc., Warrington, USA; 1 mg mL^−1^, DNA:PEI = 1:2.5) was prepared, vortexed and incubated at 20 °C for 20 min before use. HEK‐293 cells were seeded 15 h prior to transfection at a density of 1.2 × 10^5^ cells mL^−1^ in 24 well‐plates. After careful addition of 200 µL of transfection mixture, the plates were centrifuged at 200x *g* for 1 min and incubated under the above conditions for 12 h. Then, the supernatant from the transfected plates was replaced with fresh DMEM supplemented with FBS and penicillin‐streptomycin. The transfected cells were induced with DC current and after 24 h, the supernatant was collected for analysis of SEAP or VEGFA.

### Generation of a Stable Cell Line

HEK‐293 cells were seeded at a density of 1.2 ×10^5^ cells per well in 6‐well plates. The cells were co‐transfected with pJH1101 (200 ng) (ITR‐P_hCMV_‐*NRF2*‐pA:P_RPBSA_‐*ECFP*‐P2A‐*PuroR*‐pA‐ITR), pJH1054 (800 ng) (ITR‐PhCMV‐KEAP1‐P2A‐BlastR‐pA‐ITR), and pPGR 35 (400 ng) (ITR‐P_DART4_‐*SEAP*‐P2A‐*VEGFA*:P_hCMV_‐*ZeoR*‐P2A‐*mRuby*‐pA‐ITR), as well as pJH42 (P_hCMV_‐SB100X‐pA) (200 ng) which provides constitutive expression of hyperactive Sleeping Beauty transposase. The above vectors and 9.6 µL of PEI were mixed in 500 µL of reduced‐serum Opti‐MEM media and the transfection cocktail was vortexed and incubated at 20 °C for 20 min prior to transfecting the cells as described above. The HEK cells were incubated with the transfection mixture for 16 h followed by selection and clonal expansion in cell culture medium containing 2 µg mL^−1^ puromycin, 10 µg mL^−1^ blasticidin, and 100 µg m L^−1^ zeocin. The cells were cultured in an antibiotic resistance medium for 2 weeks, and stable clones of HEK_DART‐VEGFA_ were selected based on fluorescence intensity using a fluorescence‐activated cell sorter (FACSAria Fusion Cell Sorter, Becton Dickinson, New Jersey, USA). For the FACS procedure, a polyclonal population of engineered HEK_DART‐VEGFA_ cells (1 × 10^6^ cells m L^−1^) was suspended in FACS sorting buffer (PBS with 0.2% FBS and 2 mM EDTA) and collected using a 5 ml polystyrene tube with a cell‐strainer cap (35 µm, Falcon #352 235). The cells were kept on ice before the experiment. The HEK_DART‐VEGFA_ cells were sorted into 3 different sub‐populations, positive for mRuby, positive for ECFP, and positive for both mRuby and eCFP. Stable clones expressing both mRuby and eCFP were selected and cultured in resistance medium to maintain their transgenicity. The clones were periodically checked for contamination with mycoplasma or bacteria. FlowJo 10.5 software was used to analyze the FACS data.

### Analytical Assays

Colorimetric assay was used to quantify SEAP levels in both induced and uninduced cell culture media. The cell culture supernatant was inactivated at 65 °C for 30 min. The SEAP analysis cocktail was prepared using 80 µL of 2x SEAP assay buffer (20 mm homoarginine, 1 mm MgCl_2_, 21% diethanolamine, pH 9.8) and 20 µL of SEAP substrate solution (120 mm p‐nitrophenyl phosphate, cat. no. AC128860100, ThermoFisher Scientific, USA). The SEAP analysis cocktail and the heat‐inactivated cell culture supernatant were mixed in a ratio of 1:0.8 just prior to the experiment and absorbance readings were taken over a 30‐minute period at 405 nm and 37 °C using a plate reader (Tecan SPARK Reader; Tecan, Männedorf, Switzerland). The SEAP levels were quantified from a standard curve. **ELISA**. Supernatant collected from cell culture medium was evaluated for the expression of VEGFA using VEGFA ELISA Kit (Abcam, catalog no. ab119566). To switch between “On” and “Off” systems, the cell culture medium was replaced to initiate the switching. After switching, expression of the secreted protein was analyzed after 24 h. However, in the kinetic expression studies, the cell culture medium was not replaced throughout the experimental period. To determine viability, the cells were incubated for 2 h in fresh cell culture medium (DMEM supplemented with FBS and PS) containing resazurin (50 µg mL^−1^, cat. no. R7017, Sigma‐Aldrich, Saint Louis, MO, USA), under standard conditions (85% relative humidity, 5% CO_2_, 37 °C). After incubation for 2 h, absorbance readings were taken at 540/590 nm using a plate reader (Tecan SPARK Reader, Männedorf, Switzerland). **Fluorescence microscopy**. The cells were washed three times with 1x PBS and fixed using 4% paraformaldehyde solution in 1x PBS (pH 7.4) for 25 min at 37 °C.^[^ ^40^ ^]^ The cells were again washed three times with 1x PBS, and the cytoskeleton was stained with phalloidin‐rhodamine (1x dilution, Abcam, Boston, USA, cat. no. ab235138, lot. no. GR3408497‐2) for 25 minutes at 25 °C. After another three washes in 1x PBS, the nuclei were stained with SPY 650–DNA (1× dilution, Spirochrome, Thurgau, Switzerland, cat. no. SC501) for 45 minutes at 25 °C. Excess dye was removed by washing the cells twice with 1× PBS. Fluorescence microscopy was conducted using a Zeiss LSM 980 fluorescence microscope (Carl‐Zeiss, Oberkochen, Germany; phalloidin rhodamine excitation (Ex)/emission (Em) 546/575 nm, Spy 650–DNA Ex/Em 652/674 nm). For live cell imaging (live‐dead assay), cells were washed with 1× PBS (1–3 times) and stained with a live‐dead assay kit (4 mm Calcein AM, 2 mM EthD‐1, Invitrogen, ThermoFisher Scientific, Oregon, USA, cat. No. L3224, lot. no. 2 261 453) for 20 min under normal cell culture conditions. Following incubation, cells were washed three times with 1× PBS and imaged at Ex/Em 450/490 nm (green, Calcein AM) and Ex/Em 510/560 nm (red, EthD‐1) using a Zeiss LSM 980 fluorescence microscope. To assess cell internalization, HDF cells seeded on the VOLT scaffold were fixed using 4% paraformaldehyde after 3 days of incubation (85% relative humidity, 5% CO_2_, 37 °C) and stained with phalloidin‐rhodamine (1×) as described before. The samples were further stained with DAPI (1 µg mL^−1^) (Ex/Em –359 nm/457 nm) at room temperature for 10 min and washed thoroughly with 1× PBS (5 times). Finally, the samples were imaged with a Zeiss LSM 980 fluorescence microscope, and Z‐stack acquisition (z‐step size – 0.35 µm) was performed using a 20× objective with an Airyscan detector. All micrographs underwent digital processing using ZEN Blue software (Carl‐Zeiss, Oberkochen, Germany; version 3.5) and Imaris (version 10.2.0). Electron microscopy. For FESEM, the samples were cut into squares (0.5 cm × 0.5 cm) and vacuum‐dried to remove residual solvent. Then the samples were sputter‐coated with platinum (3 – 3.5 nm) using a LEICA EM ACE 600 to avoid charging. The coated samples were examined using a high‐resolution ZEISS MERLIN GEMINI II (CARL ZEISS, Germany) scanning electron microscope.

### Borophene Synthesis and Characterization

Borophene was synthesized by sonochemical liquid‐phase exfoliation of boron. In this procedure, a predetermined quantity of boron powder (CAS no. – 7440‐42‐8, Product no.–1.12070, Sigma) was dispersed in 50 mL of acetone (CAS no. – 67‐64‐1, Product no.–179 124, Sigma) and ultrasonicated using a probe sonicator operating at 30 kHz (model: Rivotek Ultrasonic Sonicator, Rivotek, India) for 24 h. The sonicated solution was centrifuged at 5000 rpm for 10 min (model: REMI CPR‐30 PLUS, Remi Group, India), and the supernatant containing borophene was collected. The obtained borophene nanosheets were characterized using TEM, AFM, and XPS for validation. The solution was stored at room temperature until required.

### Design, Fabrication, and Characterization of VOLT

Borophene was dispersed in chloroform (CAS no. – 67‐66‐3, Product no.–102 445, Merck) at various concentrations (0.5, 1, 5, 10 mg mL^−1^) by sonication for 15 min using a probe sonicator operating at 30 kHz (model: Rivotek Ultrasonic Sonicator, Rivotek, India), followed by continuous stirring at 600 rpm for 1 h. The hierarchical design of the micro/macro architecture of VOLT was achieved by electrospinning nanofibers of borophene‐PCL on top of the 3D‐printed borophene‐PCL honeycomb macro‐structure. The honeycomb‐shaped 3D borophene macroporous scaffolds were created using a 3D printing method controlled by computer‐aided design (CAD) software and operated through a 3D printer (Envisiontec, 3D Bioplotter, Germany). The 3D printing ink was formulated by uniformly dissolving 20 wt.% of polycaprolactone (average M_n_ 80000, Product no.–440 744, Sigma) in borophene‐chloroform (1 mg mL^−1^) solution. The ink was homogenized in a horizontal rolling mixer at 100 rpm for 1 h followed by brief centrifugation at 2000 rpm for 3 min to remove any bubbles prior to the 3D printing procedure. The resulting borophene‐PCL printing ink was carefully loaded into the printing tube to avoid bubble formation and extruded through a nozzle with a diameter of 400 µm under the control of the Envisiontec software. The dosing pressure was regulated within the range of 1.5−2.5 bar, and the printing speed was set at 10 mm s^−1^ at room temperature. The fabricated rectangular honeycomb structure on a metallic stage solidified after 1 h as the chloroform solvent evaporated from the printing ink. Any remaining trapped solvent was subsequently removed through vacuum drying at 40 °C for 24 h before moving to the next step. Nanofibers of borophene were prepared by electrospinning a solution of borophene and PCL in chloroform, which was prepared by directly dissolving 10 wt.% polycaprolactone (average M_n_ 80000, Product no.–440 744, Sigma) in the above borophene‐chloroform solution. The resulting solution was continuously stirred overnight at 300 rpm to obtain a homogeneous mixture. For the electrospinning process, the solution was placed in a 2.5 mL syringe equipped with a 24‐gauge blunt needle. A syringe pump was employed to maintain a constant feed rate of 4 mL per hour. Electrospinning was conducted at a voltage of 22 kV, with the needle connected to the positive output. The previously prepared 3D‐printed honeycomb structures were attached to an aluminum foil‐covered grounded collector using double‐sided tape. The distance between the collector and the needle tip was set at 15 cm and continuous electrospinning was performed for 1 h to prepare the nanofiber‐decorated VOLT matrix. 1 mg mL^−1^ concentration of borophene depicted formation of bead‐free smooth nanofibrous architecture and was selected to construct the final bi‐layered VOLT matrix. The VOLT matrix was dried overnight at room temperature and further dried in a vacuum desiccator to remove any entrapped solvent. Microstructural analysis of the VOLT matrix was performed using FESEM. The presence of borophene was confirmed using XPS, and electrical characterization was performed by I‐V analysis.

### Characterization of VOLT

In vitro degradation of the VOLT matrix was monitored for 16 weeks at 37 °C in the presence of lysozyme. Samples were incubated in 1× PBS (pH 7.4) containing 0.5 mg mL^−1^ lysozyme and washed at pre‐defined intervals using distilled water. The samples were weighed before (W_i_) and at pre‐defined intervals, the samples were weighed (W_d_) after freeze‐drying, and the % weight loss was calculated using Equation 1. **Swelling test**. Fluid absorption and retention capacity of the VOLT samples were assessed under physiological conditions at 37 °C by immersing them in 1x PBS (pH 7.4). The dry weight of the samples (W_si_) was measured at the start of the experiment and at pre‐defined time points the excess fluid on samples was carefully removed and the samples were weighed again (W_sf_). The % swelling was determined according to Equation 2. Protein adsorption studies. Protein adsorption was evaluated using the bicinchoninic acid (BCA) assay. The samples were equilibrated in 1× PBS for 24 h, and then incubated in a solution of BSA (1 mg mL^−1^ in 1× PBS) for pre‐defined periods. Post incubation, the samples were rinsed with 1× PBS, incubated in 2 wt.% sodium dodecyl sulfate (SDS) for one hour, and incubated in BCA working solution at 37 °C for 30 min. Then, the absorbance was measured at 562 nm and the protein absorbed on the samples was quantified using a standard curve. The samples for the above studies (degradation kinetics, swelling studies, and protein adsorption) were all cut into squares of similar weight to ensure consistency. Mechanical Testing. Tensile strength testing was performed using an H25KS universal testing machine (Hounsfield, UK) with a 100 N load cell. Vertically mounted samples were tested at a cross‐head speed of 5 mm min^−1^ until the failure point, maintaining a gauge length of 40 mm. Following ASTM D638‐5 standards, the samples were shaped into rectangular strips of dimensions, 60 × 10 mm (length x width) with an approximate thickness of 400 µm.

(1) $$\%Weight\;Degraded=\frac{Wi-Wd}{Wi}\;\;x\;100$$

(2) $$\%\mathrm{Swel}\mathrm{ling}=\frac{\mathrm{Wsf}-\mathrm{Wsi}}{\mathrm{Wsi}}\;\;x\;100$$

### Cell Seeding on VOLT

The VOLT matrix was cut into squares, 10 mm × 10 mm, which was fixed in a 24‐well plate using O‐rings (ø9 mm / ø5.3 mm x 5 mm). The O‐ring served to stabilize the VOLT matrix on the plate and to ensure uniform electrical stimulation of the whole matrix (Figure 4B). The VOLT matrix and the O‐ring were sterilized by incubation in 70% ethanol under UV irradiation for 30 min, then washed 5 times with 1x PBS to remove any traces of ethanol. The plate was incubated at 37 °C in DMEM cell culture medium supplemented with 10% FBS and 1% PS for 24 h prior to cell seeding. On the following day, the cell culture medium was replaced and 1 × 10^5^ cells mL^−1^ was seeded directly top of the VOLT matrix. The cell‐seeded VOLT matrix was incubated at 37 °C in DMEM cell culture medium supplemented with 10% FBS and 1% PS for 24 h prior to further evaluation and characterization. For comparison, cells were seeded at the same concentration in 24‐well plates and were incubated under identical conditions. The VOLT matrix seeded with HEK_DART‐VEGFA_ cells was termed VOLT_VEGFA_. To assess long‐term cell viability, 6 × 10^4^ cells mL^−1^ were seeded onto the VOLT matrix, and the metabolic activity of the cells was measured at specified time‐points by means of resazurin assay.

### Electrical Stimulation and Characterization

Two L‐shaped parallel platinum electrodes (0.5 mm) separated by 6 mm (previously sterilized with 70% ethanol under UV irradiation and dried) were connected to a DC power source to electro‐stimulate the system (Figure 4b). The electrical conductivity of synthesized borophene was measured across a pair of platinum electrodes connected to a Keithley 2636B source measurement unit (Tektronix, Beaverton, USA). To characterize VOLT, the matrix was directly attached to the source measurement unit using a pair of smooth crocodile clips. In both cases, the *I–V* characteristics were evaluated over a scanning voltage range of +1 V/−1 V. We also measured the electrical resistance of the samples by means of four‐probe measurements of 0.5 mm thick VOLT matrices using the above Keithley instrument. The conductivity of the VOLT matrix was calculated and reported as the mean of *n* = 5 samples.

### Animal experiments

The study was approved by the institute ethical committee (IEC) of the Indian Institute of Technology Kharagpur, India (IE‐1/SN‐ChE/3.19). Wistar rats were used to prepare the wound healing model. The dorso‐ventral part of the animals was shaved, cleaned, and rinsed with betadine solution prior to application and stimulation. **Wound healing in Wistar rats**. The VOLT matrices were cut into squares (2.5 cm^2^ × 2.5 cm^2^), sterilized, and seeded with HEK_DART‐VEGFA_ cells as described above and are termed as VOLT_VEGFA_. Prior to application, the VOLT_VEGFA_ samples were incubated at 37 °C, 5% CO_2_ in standard cell culture medium (DMEM, 10% FBS, and 1% PS) for 3 days. The Wistar rats were anesthetized with 4% isoflurane and then injected with ketamine hydrochloride (90 mg kg^−1^) and xylazine hydrochloride (10 mg kg^−1^). Anaesthesia was maintained with 2% isoflurane during surgery. A 2 cm × 2 cm subcutaneous wound bed was created, penetrating to the level of the subcutaneous panniculus carnosus. The VOLT_VEGFA_ sample was then placed on the wound bed and fixed using a skin stapler (B. Brown, Manipler AZ) and a Tegaderm dressing. The experimental groups were stimulated with 5V DC bias for 20s using platinum‐coated needles (Figure 5A), daily until the 5th day then again on the 7th and 10th day while the non‐stimulated groups were used as controls. In a separate experiment to evaluate long‐term expression of VEFGA, VOLT_VEGFA_ was implanted subcutaneously on the dorso‐ventral side of rats. The point of incision was closed using surgical sutures and a skin stapler. The implant was stimulated with 5 V DC bias for 20 s at pre‐determined time‐points, and blood samples were collected from the tail vein in Microtainer tubes 24 h thereafter. Serum was separated by centrifugation at 6000× *g* for 10 min at 4 °C (Becton Dickinson, cat. no. 365 967, New Jersey, USA). The circulating level of VEFGA in blood samples was measured using ELISA according to manufacturer's protocol (VEGFA ELISA Kit, Abcam, catalog no. ab119566). Serum levels of inflammatory cytokines were profiled using IL‐6 (cat. no. ERA31RB) and TNF‐α (cat. no. Catalog # BMS622) ELISA kits (both from Thermo Fisher).

### Tissue Collection, Histology, and Immunohistochemistry

The effect of electrostimulation‐guided release of VEGFA on wound healing kinetics was monitored regularly and tissue samples were collected on days 5, 10, and 15 for histological and immunohistochemical analysis. At each time point, animals were euthanized, and the length of the healed wound bed was documented using a Canon EOS 750D digital camera positioned at a fixed distance. The dimensions of the healed region were assessed using Image J software (NIH, USA), and the wound closure percentage was computed using the formula:

(3) $$Wound\;Closure\;Rate\;\;\left(WCr\%\right)=\frac{Wi-Wn}{Wi}\;x\;100$$

*Wi* and *Wn* represent the initial and final length of the wound bed, recorded on the day of tissue collection. After euthanasia, the regenerated tissue was excised and preserved in 10% formalin phosphate‐buffered solution (Sigma) before being embedded in paraffin blocks for staining. Thin sections (4 µm) were prepared using a rotary microtome (RM2135, Leica, Germany) and stained with hematoxylin and eosin (H&E, Sigma) or Masson's trichrome (MT, Sigma) staining solution according to the manufacturer's protocols. The % re‐epithelialization of the healed tissue was evaluated from H&E‐stained tissue samples using Image J software by applying the following equation, where Sr represents the re‐epithelialized area and Sw represents the remaining wound area:

(4) $$Re-\mathrm{epithelialization}\%=\frac{\mathrm{Sr}-\mathrm{Sw}}{\mathrm{Sr}}\;\;x\;100$$

For each time point, the % re‐epithelialization was calculated from *n* = 10 independent micrographs randomly collected from all four samples. In addition, immunohistochemical analysis of microtome sections was conducted using anti‐CD31 (Invitrogen) antibodies to examine neovascularization and blood vessel development, while anti‐COL I (Invitrogen) was used to confirm collagen deposition at given time points. The procedures were performed as per the manufacturer's instructions and the staining process utilized a chromogen from a DAB kit (BioGenex). After staining, the samples were analyzed using a Zeiss Axio Observer Z1 fluorescence microscope. Vascular density was quantified from micrographs of anti‐CD31 immuno stained samples using Image J's vascular density analysis tool. The CD31 channel was isolated and converted to grayscale. A threshold was applied to identify positive regions while generating a binary image. The total CD31‐positive area and area fraction were measured in the region of interest, and the percentage of the image occupied by CD31 staining was calculated.

### Reverse Transcription Polymerase Chain Reaction (RT‐PCR)

Expression of genes associated with re‐epithelialization and vascularization was studied in healed tissues recovered from the wound site at days 5, 10, and 15. Animals were euthanized, and the samples of healing tissue were collected and preserved in RNAlater solution (Invitrogen, US) at −80 °C. RNA was recovered using TRIzol according to the manufacturer's protocol (Thermo Fisher Scientific, Waltham, MA). cDNA was prepared using the Verso cDNA synthesis kit. Gene expression levels were quantified with PowerUp SYBR Green qPCR master mix using the Quant Studio 5 Real‐Time PCR System (Applied Biosystems). The expression levels of transforming growth factor‐β (TGF‐β), collagen III (Col III), collagen I (Col I), epidermal growth factor (EGF), IL‐6, TNF‐α and fibroblast growth factor (FGF) were evaluated with GAPDH as an internal control. The primers used in the RT‐PCR study were specific for *Rattus norvegicus* and are listed in Table S2 (Supporting Information). Each reaction was performed in triplicate, and the fold change in gene expression was calculated using the ΔΔCt method, with the expression levels in normal skin serving as the reference control. Fold change values were calculated as 2^−(ΔΔCt)^.

### Plots and Figures

All plots were prepared in GraphPad Prism 10.2.3. Schematics and figures were designed and assembled using Biorender and Microsoft PowerPoint. Figures were analyzed using Fiji. Data were recorded and managed using Microsoft Excel.

### Statistics and Reproducibility

The statistical significance of differences among groups was evaluated using two‐tailed, unpaired Student's *t*‐test or one‐way ANOVA with Dunnett's multiple comparisons test (as specified in the figure legends) implemented in GraphPad Prism 10.2.1. Homogeneity of variances and normal distribution of values were conducted before performing the statistical comparison tests. Differences are considered statistically significant at *p* < 0.05. The statistical test used, and the level of significance are reported in the figure legends. Experiments were independently repeated four times or as stated in the figure legends.

### Data Availability

The authors declare that all data generated in this study are provided within the paper and in the Supporting Information. All plasmid information is provided in Table S1. The plasmid sequences used in this study can be found online in the GenBank database under the following accession codes: pJH1003 (GenBank accession no. ON256650), pJH1004 (GenBank accession no. ON256651), pJH1005 (GenBank accession no. ON256652), pJH1054 (GenBank accession no. ON256653), pJH1101 (GenBank accession no. ON256654) and pJH1169 (GenBank accession no. ON256655). Requests for materials should be made to the corresponding author. All plasmids generated in this study are available upon request.

## Conflict of Interest

The authors declare no conflict of interest.

## Author Contributions

P.G.R. and M.F. designed the project, analyzed the results, and wrote the manuscript; P.G.R. conducted the in vitro experiments; B.P. synthesized and characterized borophene and P.G.R. fabricated and characterized the VOLT matrix; P.G.R designed the plasmids and generated the stable cell line; P.G.R. and R.R. performed the animal experiments related staining and characterization; S.D. and S.D. facilitated writing the manuscript and analyzed the results.

## Supporting information



Supporting Information

Supplemental Movie 1

## Data Availability

The data that support the findings of this study are available from the corresponding author upon reasonable request.

## References

[1] C. J. Bashor , I. B. Hilton , H. Bandukwala , D. M. Smith , O. Veiseh , Nat. Rev. Drug Discovery 2022, 21, 655. 35637318 10.1038/s41573-022-00476-6 PMC9149674

[2] Z. Zhao , X. Chen , A. M. Dowbaj , A. Sljukic , K. Bratlie , L. Lin , E. L. S. Fong , G. M. Balachander , Z. Chen , A. Soragni , Nat. Rev. Methods Primers 2022, 2, 94. 37325195 10.1038/s43586-022-00174-y PMC10270325

[3] J. Sun , W. Xie , Y. Wu , Z. Li , Y. Li , Adv. Sci. 2024, 2404190. 10.1002/advs.202404190 PMC12199592 39115981

[4] J. Garcia , J. Daniels , Y. Lee , I. Zhu , K. Cheng , Q. Liu , D. Goodman , C. Burnett , C. Law , C. Thienpont , Nature 2024, 626, 626. 38326614 10.1038/s41586-024-07018-7 PMC11573425

[5] L. Scheller , T. Strittmatter , D. Fuchs , D. Bojar , M. Fussenegger , Nat. Chem. Biol. 2018, 14, 723. 29686358 10.1038/s41589-018-0046-z

[6] D. Maity , P. Guha Ray , P. Buchmann , M. Mansouri , M. Fussenegger , Adv. Mater. 2023, 35, 2300890. 10.1002/adma.202300890 36893359

[7] T. Strittmatter , Y. Wang , A. Bertschi , L. Scheller , P. C. Freitag , P. G. Ray , P. Stuecheli , J. V. Schaefer , T. Reinberg , D. Tsakiris , Nat. Chem. Biol. 2022, 18, 1125. 35941237 10.1038/s41589-022-01095-3 PMC9512699

[8] M. Mansouri , P. G. Ray , N. Franko , S. Xue , M. Fussenegger , Nucleic Acids Res. 2023, 51, 1. 36268868 10.1093/nar/gkac916 PMC9841418

[9] S. Bose , L. R. Volpatti , D. Thiono , V. Yesilyurt , C. McGladrigan , Y. Tang , A. Facklam , A. Wang , S. Jhunjhunwala , O. Veiseh , Nat. Biomed. Eng. 2020, 4, 814. 32231313 10.1038/s41551-020-0538-5 PMC8051527

[10] C. O'Connor , E. Brady , Y. Zheng , E. Moore , K. R. Stevens , Nat. Rev. Mater. 2022, 7, 702. 35669037 10.1038/s41578-022-00447-8 PMC9154041

[11] R. M. Touyz , A. M. Briones , Hypertens. Res. 2011, 34, 5. 20981034 10.1038/hr.2010.201

[12] R. S. Apte , D. S. Chen , N. Ferrara , Cell 2019, 176, 1248. 30849371 10.1016/j.cell.2019.01.021 PMC6410740

[13] J. M. Isner , A. Pieczek , R. Schainfeld , R. Blair , L. Haley , T. Asahara , K. Rosenfield , S. Razvi , K. Walsh , J. F. Symes , Lancet 1996, 348, 370. 8709735 10.1016/s0140-6736(96)03361-2

[14] W. S. Moon , K. H. Rhyu , M. J. Kang , D. G. Lee , H. C. Yu , J. H. Yeum , G. Y. Koh , A. S. Tarnawski , Mod. Pathol. 2003, 16, 552. 12808060 10.1097/01.MP.0000071841.17900.69

[15] J. L. Terrell , T. Tschirhart , J. P. Jahnke , K. Stephens , Y. Liu , H. Dong , M. M. Hurley , M. Pozo , R. McKay , C. Y. Tsao , Nat. Nanotechnol. 2021, 16, 688. 33782589 10.1038/s41565-021-00878-4

[16] J. Huang , S. Xue , P. Buchmann , A. P. Teixeira , M. Fussenegger , Nat. Metab. 2023, 5, 1395. 37524785 10.1038/s42255-023-00850-7 PMC10447240

[17] K. Krawczyk , S. Xue , P. Buchmann , G. Charpin‐El‐Hamri , P. Saxena , M.‐D. Hussherr , J. Shao , H. Ye , M. Xie , M. Fussenegger , Science 2020, 368, 993. 32467389 10.1126/science.aau7187

[18] P. G. Ray , D. Maity , J. Huang , H. Zulewski , M. Fussenegger , Nat. Commun. 2023, 14, 3151. 37258547 10.1038/s41467-023-39015-1 PMC10232489

[19] B. Pratihar , O. Roy , A. Jana , S. De , J. Colloid. Interface Sci. 2024, 661, 279. 38301466 10.1016/j.jcis.2024.01.188

[20] Y. V. Kaneti , D. P. Benu , X. Xu , B. Yuliarto , Y. Yamauchi , D. Golberg , Chem. Rev. 2021, 122, 1000. 34730341 10.1021/acs.chemrev.1c00233

[21] B. Peng , H. Zhang , H. Shao , Y. Xu , R. Zhang , H. Zhu , J. Mater. Chem. C 2016, 4, 3592.

[22] H. Zhou , Y. Cai , G. Zhang , Y.‐W. Zhang , npj 2D Mater. Appl. 2017, 1, 14.

[23] P. Ranjan , T. K. Sahu , R. Bhushan , S. S. Yamijala , D. J. Late , P. Kumar , A. Vinu , Adv. Mater. 2019, 31, 1900353. 10.1002/adma.201900353 31044470

[24] C. Zhong , X. Li , P. Yu , Commun. Phys. 2024, 7, 38.

[25] B. Feng , J. Zhang , R.‐Y. Liu , T. Iimori , C. Lian , H. Li , L. Chen , K. Wu , S. Meng , F. Komori , Phys. Rev. B 2016, 94, 041408.

[26] B. Feng , O. Sugino , R.‐Y. Liu , J. Zhang , R. Yukawa , M. Kawamura , T. Iimori , H. Kim , Y. Hasegawa , H. Li , Phys. Rev. Lett. 2017, 118, 096401. 28306312 10.1103/PhysRevLett.118.096401

[27] B. Feng , J. Zhang , S. Ito , M. Arita , C. Cheng , L. Chen , K. Wu , F. Komori , O. Sugino , K. Miyamoto , Adv. Mater. 2018, 30, 1704025. 10.1002/adma.201704025 29171690

[28] H. Li , L. Jing , W. Liu , J. Lin , R. Y. Tay , S. H. Tsang , E. H. T. Teo , ACS Nano 2018, 12, 1262. 29378394 10.1021/acsnano.7b07444

[29] S. K. Ghorai , T. Roy , S. Maji , P. G. Ray , K. Sarkar , A. Dutta , A. De , S. Bandyopadhyay , S. Dhara , S. Chattopadhyay , Chem. Eng. J. 2022, 429, 132179.

[30] S. K. Ghorai , A. Dutta , T. Roy , P. Guha Ray , D. Ganguly , M. Ashokkumar , S. Dhara , S. Chattopadhyay , ACS Appl. Mater. Interfaces 2022, 14, 28455. 35715225 10.1021/acsami.2c01657

[31] D. M. da Silva , N. Barroca , S. C. Pinto , Â. Semitela , B. M. de Sousa , P. A. Martins , L. Nero , I. Madarieta , N. García‐Urkia , F.‐J. Fernández‐San‐Argimiro , Chem. Eng. J. 2023, 472, 144980.

[32] G. Zhao , H. Qing , G. Huang , G. M. Genin , T. J. Lu , Z. Luo , F. Xu , X. Zhang , NPG Asia Mater. 2018, 10, 982.

[33] L. Yan , L. Wang , J. Wu , Y. Wu , X. Zhu , Q. Mei , Y. Song , Y. Liu , L. Zhang , J. Ai , NPJ Regener. Med. 2022, 7, 52. 10.1038/s41536-022-00236-5 PMC9481528 36114211

[34] S. W. Chen , S. M. Huang , H. S. Wu , W. P. Pan , S. M. Wei , C. W. Peng , I. C. Ni , B. T. Murti , M. L. Tsai , C. I. Wu , Adv. Sci. 2022, 9, 2201507. 10.1002/advs.202201507 PMC9353498 35657078

[35] P. Das , S. Ganguly , P. K. Marvi , M. Sherazee , S. R. Ahmed , X. Tang , S. Srinivasan , A. R. Rajabzadeh , Adv. Funct. Mater. 2024, 2314520. 10.1002/adma.202409819 PMC11602684 39394767

[36] T. A. Mollner , P. G. Isenegger , B. Josephson , C. Buchanan , L. Lercher , D. Oehlrich , D. F. Hansen , S. Mohammed , A. J. Baldwin , V. Gouverneur , Nat. Chem. Biol. 2021, 17, 1245. 34725511 10.1038/s41589-021-00883-7 PMC8604732

[37] S. L. Hirsh , D. R. McKenzie , N. J. Nosworthy , J. A. Denman , O. U. Sezerman , M. M. Bilek , Colloids Surf., B 2013, 103, 395. 10.1016/j.colsurfb.2012.10.039 23261559

[38] D. W. Leung , G. Cachianes , W.‐J. Kuang , D. V. Goeddel , N. Ferrara , Science 1989, 246, 1306. 2479986 10.1126/science.2479986

[39] S.‐W. Kim , H. Kim , H.‐J. Cho , J.‐U. Lee , R. Levit , Y.‐s. Yoon , J. Am. Coll. Cardiol. 2010, 56, 593. 20688215 10.1016/j.jacc.2010.01.070 PMC2917842

[40] P. Guha Ray , P. Pal , P. K. Srivas , P. Basak , S. Roy , S. Dhara , ACS Appl. Bio Mater. 2018, 1, 985. 10.1021/acsabm.8b00169 34996140

[41] A. Dove , Nat. Biotechnol. 2002, 20, 339. 11923834 10.1038/nbt0402-339

[42] G. Orive , R. M. Hernández , A. R. Gascón , R. Calafiore , T. M. Chang , P. D. Vos , G. Hortelano , D. Hunkeler , I. Lacík , A. J. Shapiro , Nat. Med. 2003, 9, 104. 12514721 10.1038/nm0103-104

[43] H. K. Kleinman , G. R. Martin , Semin. Cancer Biol. 2005, 378. 15975825 10.1016/j.semcancer.2005.05.004

[44] Q. Zhou , B. D. Johnson , R. J. Orentas , Cell. Immunol. 2007, 245, 91. 17543914 10.1016/j.cellimm.2007.04.004 PMC1949498

[45] O. Smidsrød , G. Skja , Trends Biotechnol. 1990, 8, 71. 1366500 10.1016/0167-7799(90)90139-o

[46] B. Baudin , A. Bruneel , N. Bosselut , M. Vaubourdolle , Nat. Protoc. 2007, 2, 481. 17406610 10.1038/nprot.2007.54

