# Supplementary material for: Skin‐Integrated Electrogenetic Regulation of Vasculature for Accelerated Wound Healing
Source: Adv Sci (Weinh). 2025 Jan 10;12(9):2412257. doi: 10.1002/advs.202412257 (PMC11884547; doi:10.1002/advs.202412257)
Supplement: Supplementary file 1 — Supporting Information [file ADVS-12-2412257-s001.pdf]

ADVANCED SCIENCE
Open Access

Supporting Information

for *Adv. Sci.*, DOI 10.1002/advs.202412257

Skin-Integrated Electrogenetic Regulation of Vasculature for Accelerated Wound Healing

*Preetam Guha Ray, Ragavi Rajasekaran, Bitan Pratihar, Sirshendu De, Santanu Dhara and Martin Fussenegger**

Supporting Information

Skin-Integrated Electrogenetic Regulation of Vasculature for Accelerated Wound Healing

Preetam Guha Ray[1,2], Ragavi Rajasekaran[2], Bitan Pratihar[3], Sirshendu De[3], Santanu Dhara[2], Martin Fussenegger[1,4,*]

[1]ETH Zurich, Department of Biosystems Science and Engineering, Klingelbergstrasse 48, CH-4056 Basel, Switzerland
[2]Biomaterials and Tissue Engineering Laboratory, School of Medical Science and Technology (SMST), Indian Institute of Technology Kharagpur, Kharagpur 721302, India.
[3]Department of Chemical Engineering, Indian Institute of Technology Kharagpur, Kharagpur 721302, India.
[4]Faculty of Science, University of Basel, Klingelbergstrasse 48, CH-4056 Basel, Switzerland.

[*]Corresponding author E-mail: martin.fussenegger@bsse.ethz.ch

The PDF file includes:

Figures S1 – S14

Tables S1 – S2

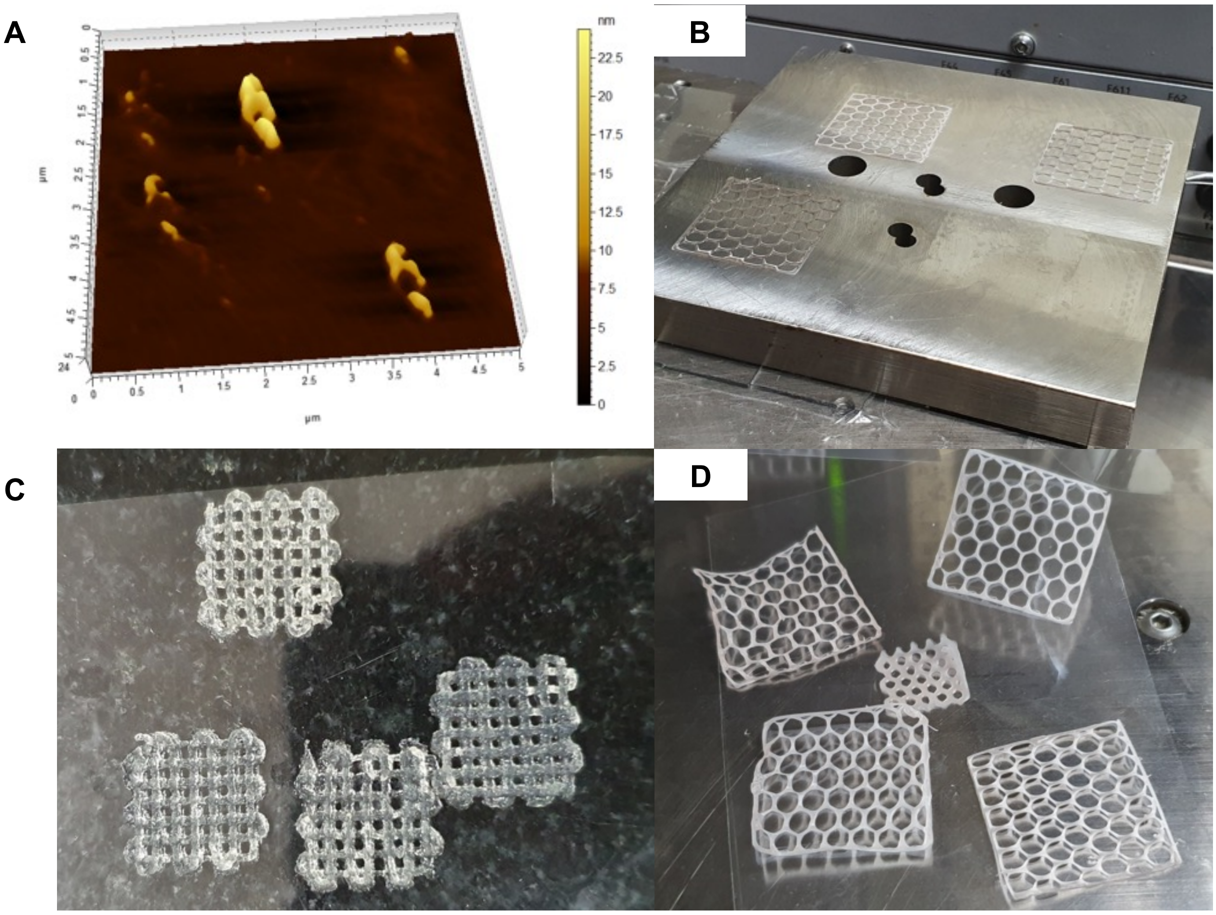

Figure S1 | (A) AFM micrograph of borophene as prepared. **(B-C)** Digital images showing 3D-printed borophene/PCL sheets prior to drying. The borophene/PCL slurry was printed in honeycomb and 0/90 angle shaped structures to demonstrate the versatility of the slurry. **(D)** Fabricated borophene honeycomb structures after drying. The honeycomb structure was chosen to provide superior mechanical properties.

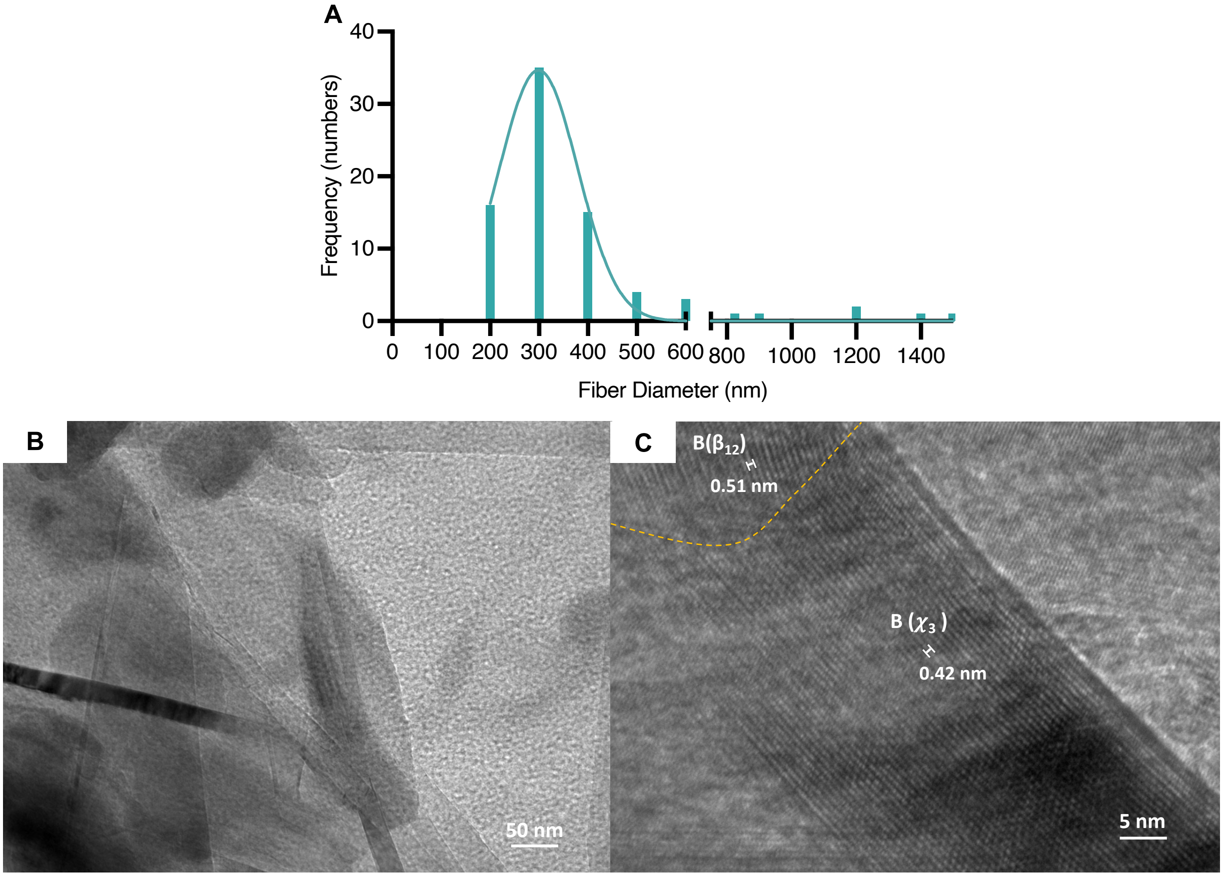

Figure S2 | Characterization of the VOLT matrix. (A) The fiber diameter distribution of the VOLT matrix calculated using Fiji and GraphPad Prism depicts a narrow Gaussian distribution. **(B-C)** The VOLT matrix was completely dissolved in chloroform/acetone and coated on a TEM grip for analysis. Transmission electron microscopy confirmed the presence of borophene in the VOLT matrix from the presence of both the β_{12} ($d_{spacing}$ – 0.51 nm) and χ_3 ($d_{spacing}$ – 0.42 nm) phases.

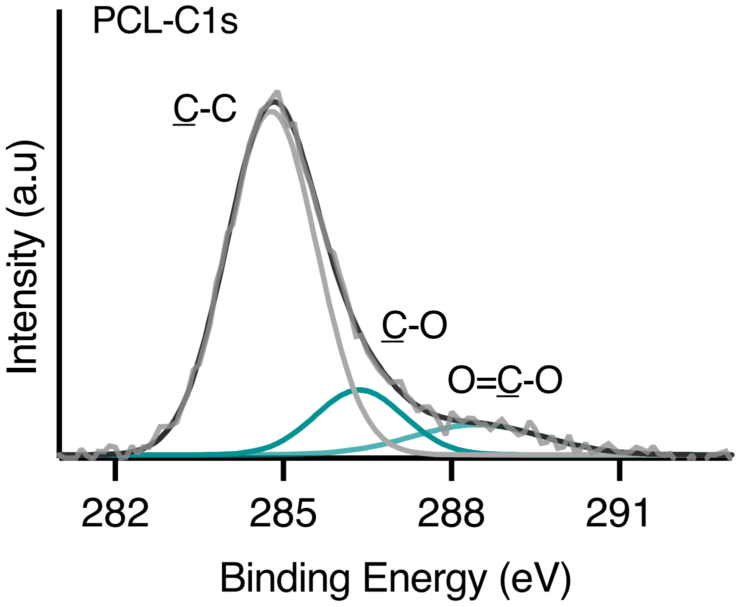

Figure S3 | XPS of PCL. The C1s XPS spectrum of PCL after deconvolution shows subpeaks at binding energies of around 284.79 eV, 286.36 eV and 288.47 eV. These are also observed in the VOLT matrix.

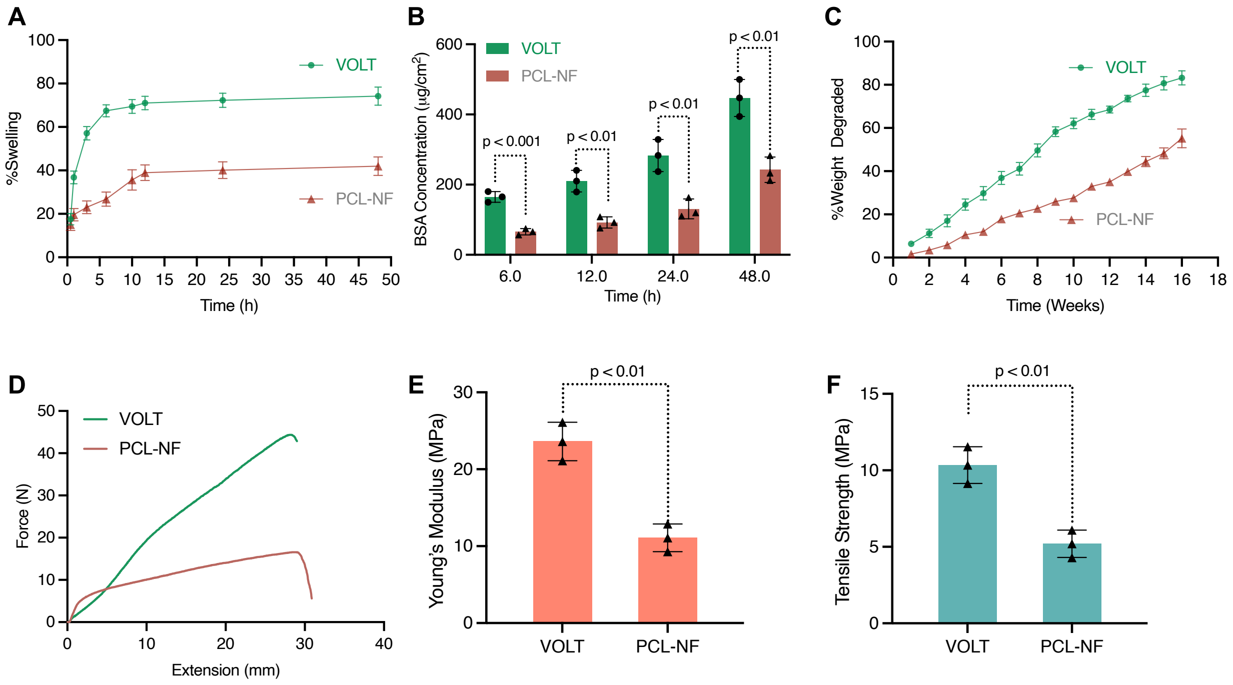

Figure S4| Characterization of VOLT. (A) and **(C)** depict % swelling and % degradation (by weight) of the VOLT matrix as compared to PCL-NF. **(B)** Kinetics of BSA adsorption by the VOLT matrix. **(D-F)** Mechanical properties of a 400 μm thick VOLT scaffold were studied using a 100 N load cell in comparison with a standard PCL-NF matrix of similar dimensions. Data are presented as mean ± SD of n = 3 independent samples. The statistical significance of differences (*p value*) was calculated using a two-tailed, unpaired Student's t-test.

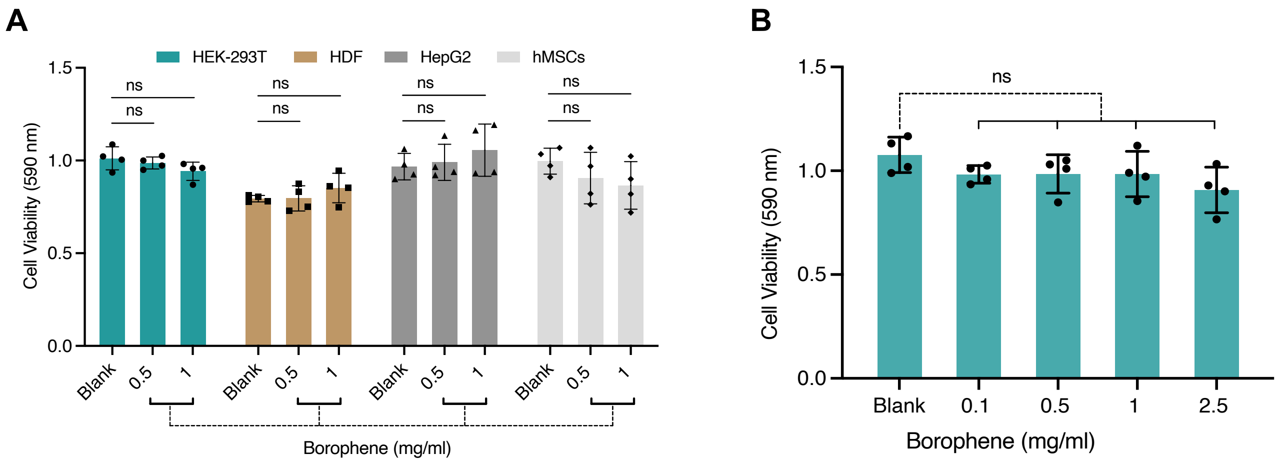

Figure S5 | Viability of adherent cells. (A) The cytotoxicity of as-prepared borophene (0.5 and 1 mg/ml) towards HEK-293T cells, human dermal fibroblasts, HepG2 cells and hMSCs cells was assessed by means of resazurin assay in 96-well plate culture. Cell viability was compared to that of untreated cells (Blank). **(B)** HEK-293T cells were exposed to various concentrations (0.1 – 2.5 mg/ml) of borophene. Data are presented as mean ± SD of n = 4 biologically independent samples. The statistical significance of differences (*p value*) was calculated using one-way ANOVA with Dunnett's multiple comparisons test.

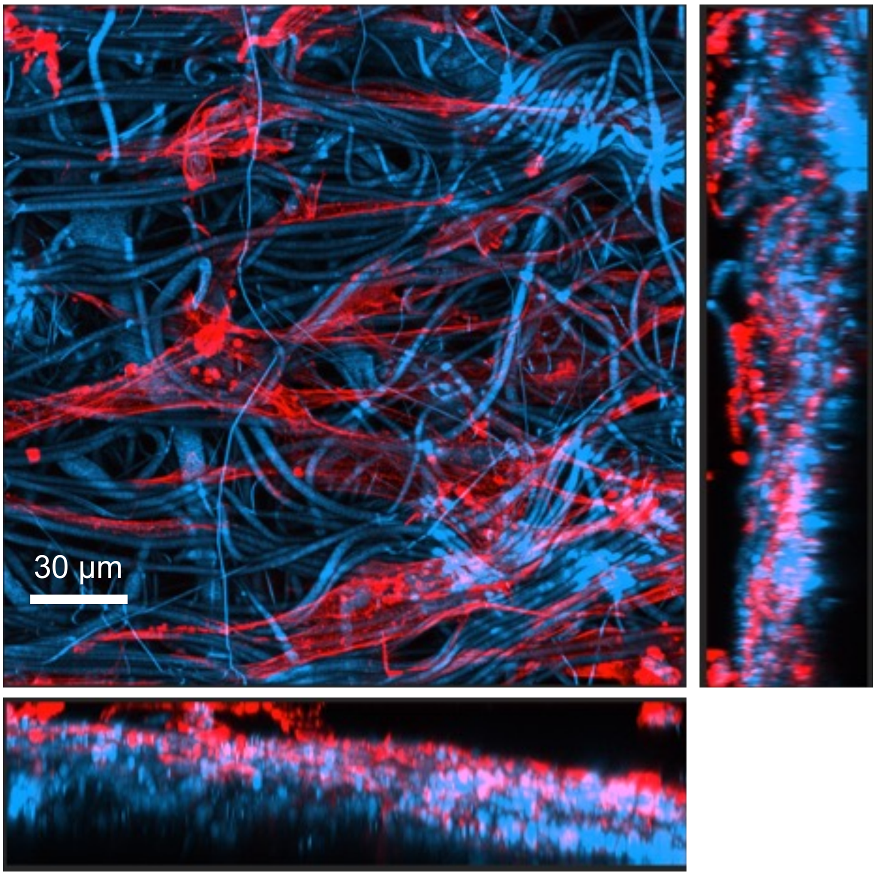

Figure S6 | 3D representation of Z-stacked fluorescence micrographs illustrating infiltration of HDF cells into the interior of the VOLT scaffold. Bottom and side bars represent cross-sectional views of the z-stack.

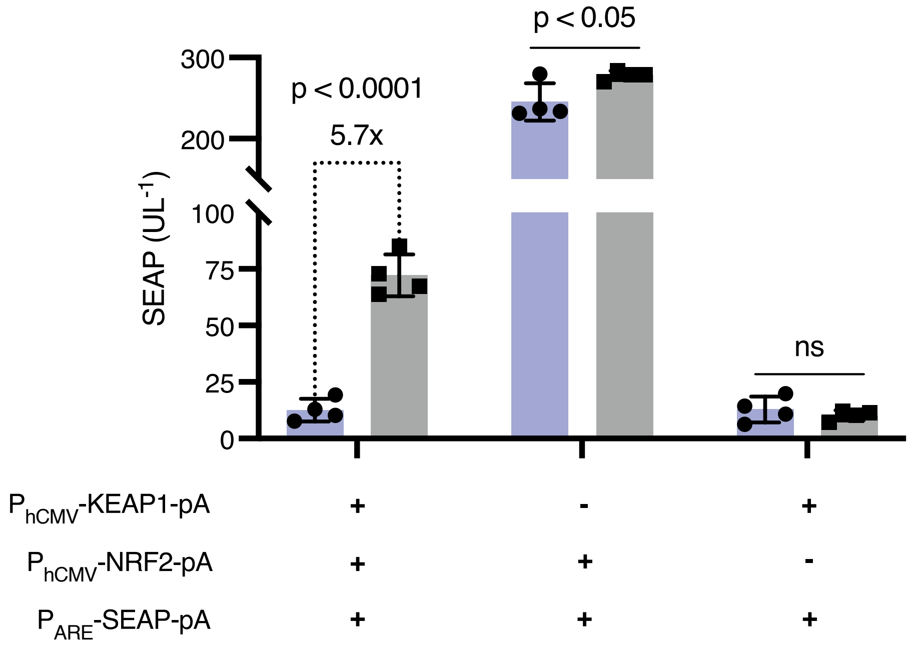

Figure S7 | Component-wise dissection of the DART system. The DART sensor was activated only in the presence of all three components. When HEK-293T cells transfected only with *NRF2*, P_{hCMV}-*NRF2*-pA and the ARE operator-reporter, O_{ARE}-$P_{hCMVmin}$, high SEAP expression was observed in the supernatant with or without electrical stimulation (DC 5 V, 20 s), presumably due to the constitutive expression of the *NRF2* gene. Data are presented as mean ± SD of n = 4 biologically independent samples. The statistical significance of differences (*p* value) was calculated using a two-tailed, unpaired Student's t-test.

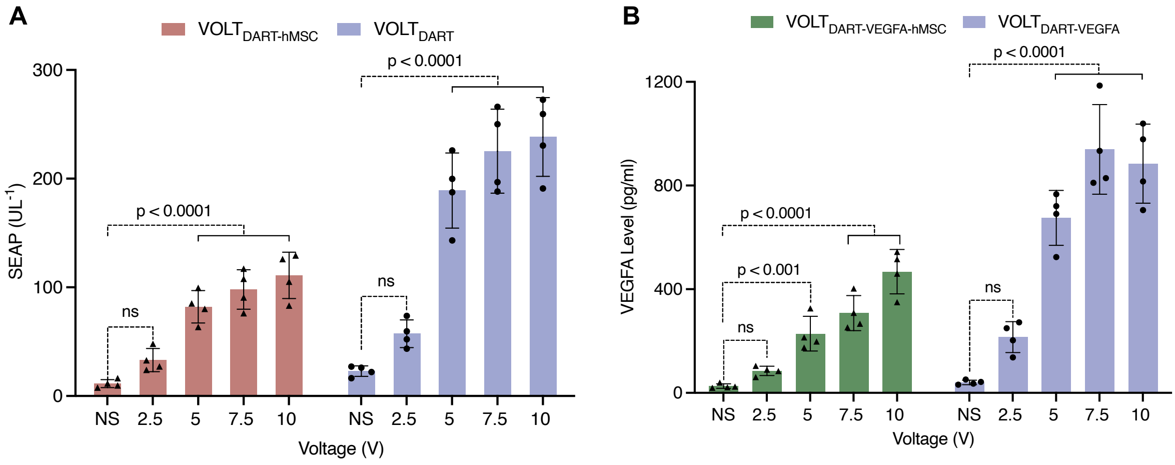

Figure S8 | The DART technology with SEAP **(A)** or VEGFA **(B)** as a reporter was transfected into HEK 293 cells ($VOLT_{DART}$ – SEAP as reporter and $VOLT_{DART\text{-}VEGFA}$ – VEGFA as reporter) and hMSCs ($VOLT_{DART\text{-}hMSC}$ – SEAP as reporter and $VOLT_{DART\text{-}VEGFA\text{-}hMSC}$ – VEGFA as reporter). The expression levels of SEAP and VEGFA after stimulation at DC 5 V for 20 s indicated that the $VOLT_{DART\text{-}HEK}$ system outperforms the $VOLT_{DART\text{-}hMSC}$ system. Data are presented as mean ± SD of n = 4 biologically independent samples. The statistical significance of differences (*p value*) was calculated using one-way ANOVA with Dunnett's multiple comparisons test.

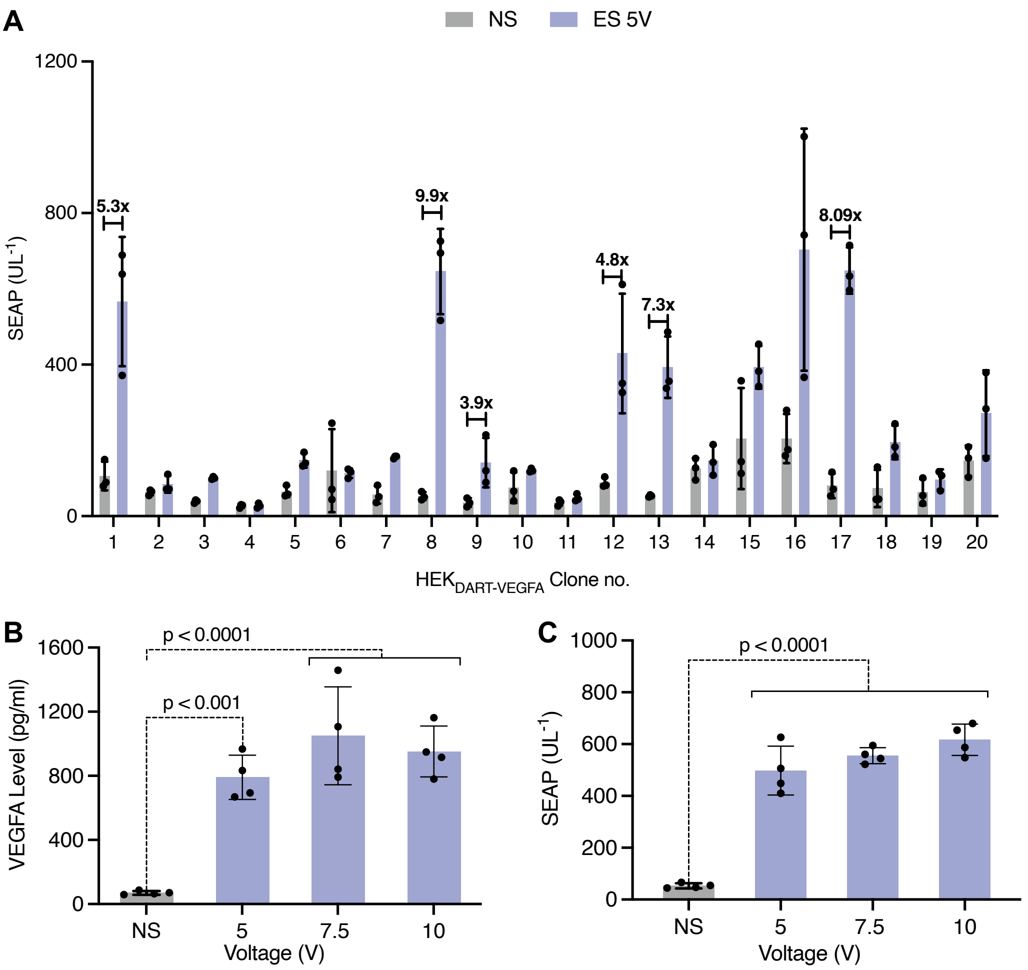

Figure S9 |Selection of stable HEK$_{DART-VEGFA}$ cell clone. (A) Clones tested for the HEK$_{DART-VEGFA}$ system. The colonies were induced by electrostimulation (DC 5 V, 20 s) and the SEAP expression was compared with the uninduced basal level. The best-in-class clone, no. 8, was selected for further studies. Data are presented as mean ± SD of n = 3 biologically independent samples. **(B-C)** Activation and specificity of stably transgenic HEK$_{DART-VEGFA}$ (colony no. 8) were checked by electro-stimulating the cells at increasing voltages (DC – 5 V, 7.5 V, 10 V for 20 s). Data are presented as mean ± SD of n = 4 biologically independent samples. The statistical significance of differences (*p value*) was calculated using one-way ANOVA with Dunnett's multiple comparisons test.

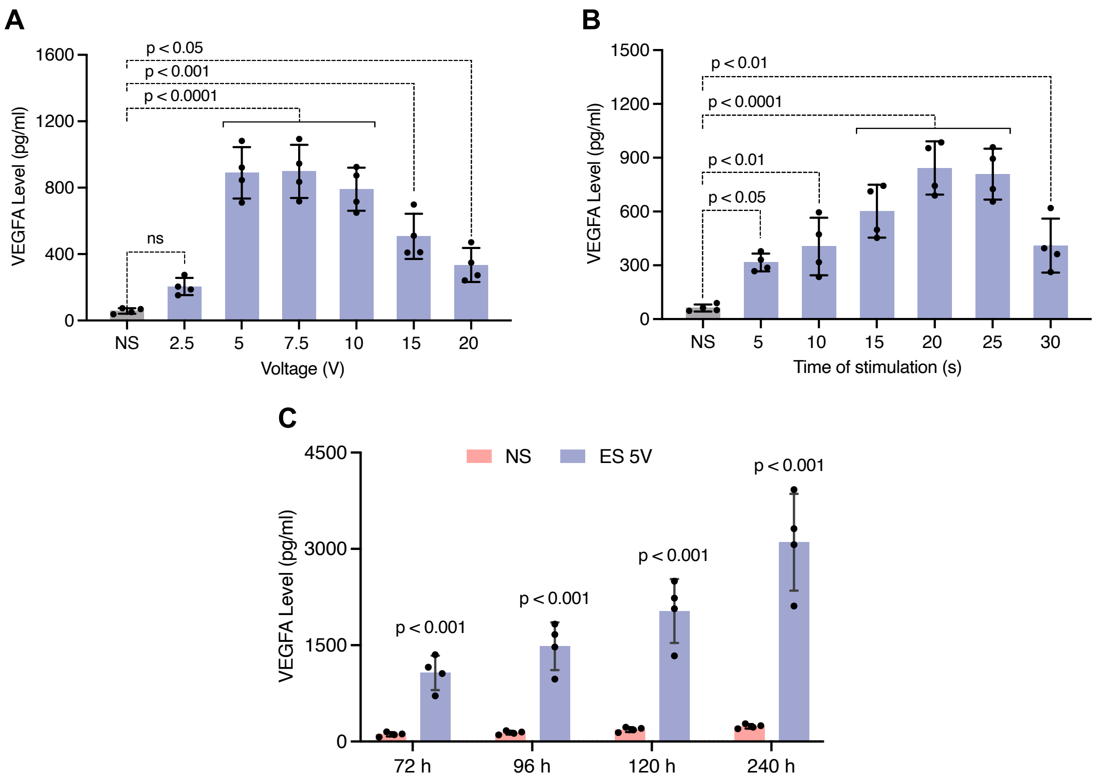

Figure S10 | (A, B) VEGF-A expression profile of the $VOLT_{VEGFA}$ system when electro-stimulated at increasing voltages (2.5 V to 20 V) for various times (5-30 s). **(C)** The kinetics of VEGF-A expression by the electro-stimulated $VOLT_{VEGFA}$ system was recorded until 240 h (10 days). Data are presented as mean ± SD of n = 4 biologically independent samples. For (A) and (B), the statistical significance of differences (*p value*) was calculated using one-way ANOVA with Dunnett's multiple comparisons test. For C, *p* values were calculated using a two-tailed, unpaired Student's *t*-test.

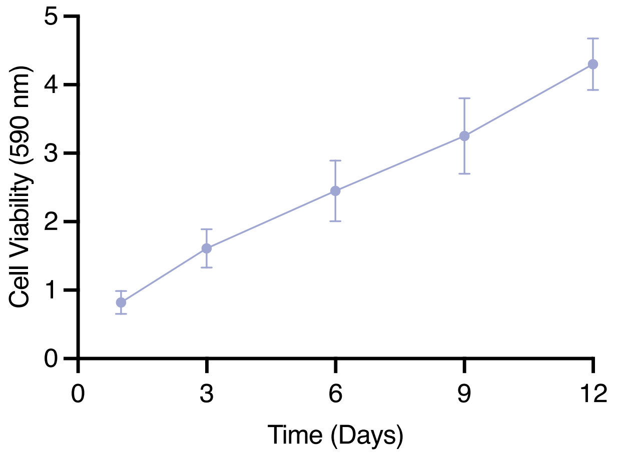

Figure S11 | Long-term viability of the engineered cells in the $VOLT_{VEGFA}$ system was confirmed by resazurin assay. Data are presented as mean ± SD of n = 4 biologically independent samples.

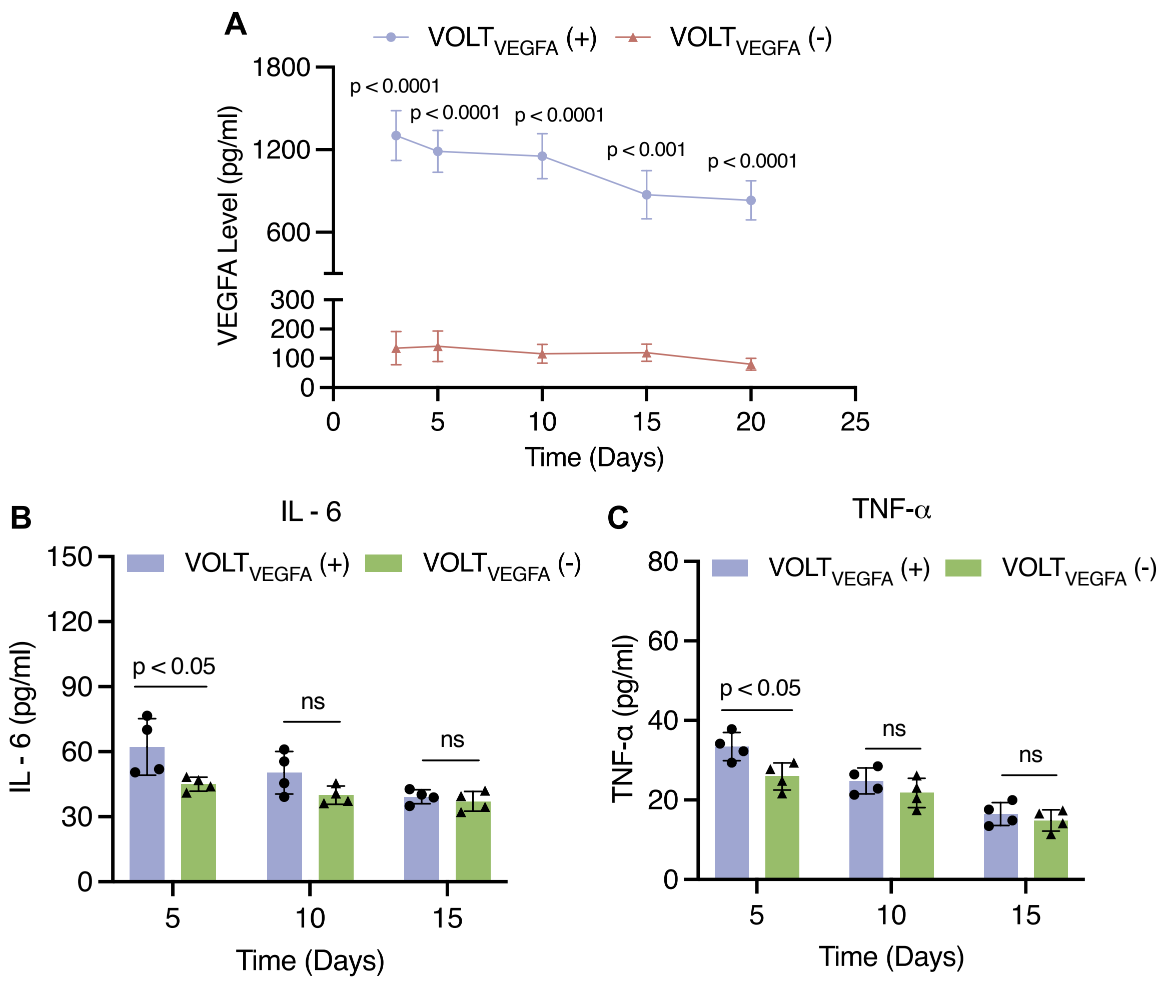

Figure S12 |(A) Electrostimulated (DC 5 V, 20 s) VEGFA expression from subcutaneously implanted VOLT$_{VEGFA}$ (+) was monitored using ELISA and compared with that of the non-stimulated VOLT$_{VEGFA}$ (-) group. **(B-C)** Levels of IL-6 and TNF-α were measured in blood samples collected at different time points. Data are presented as mean ± SD of n = 4 biologically independent samples. The statistical significance of differences (p value) was calculated using a two-tailed, unpaired Student's t-test

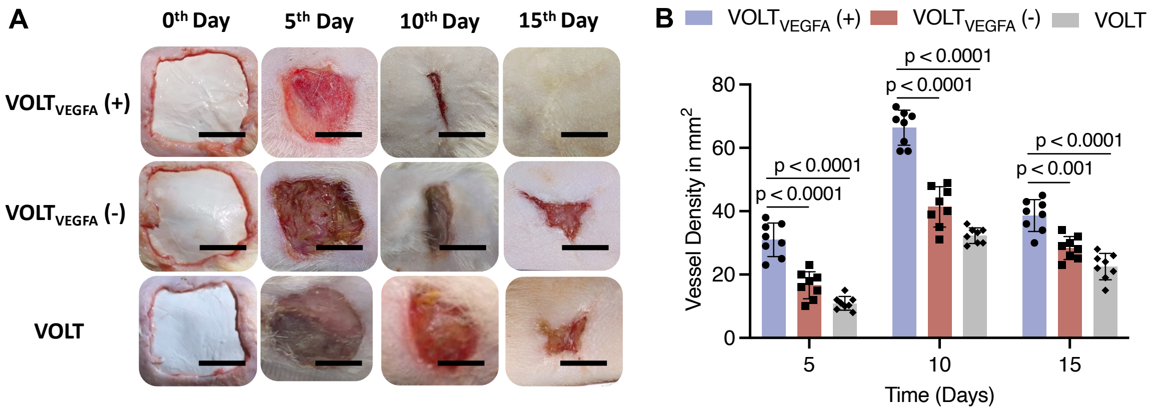

Figure S13 | (A) Digital photographs of the wound-healing process (Scale Bar: 1 cm). Vasculature in the healing tissue was evaluated from micrographs of CD31 immuno-stained samples using Image J software. The vessel density at various time-points for each group is represented in **(B)**. At each time point, vessel density was calculated from micrographs of n = 8 randomly collected from independent samples. The statistical significance of differences (*p value*) was calculated using one-way ANOVA with Dunnett's multiple comparisons test.

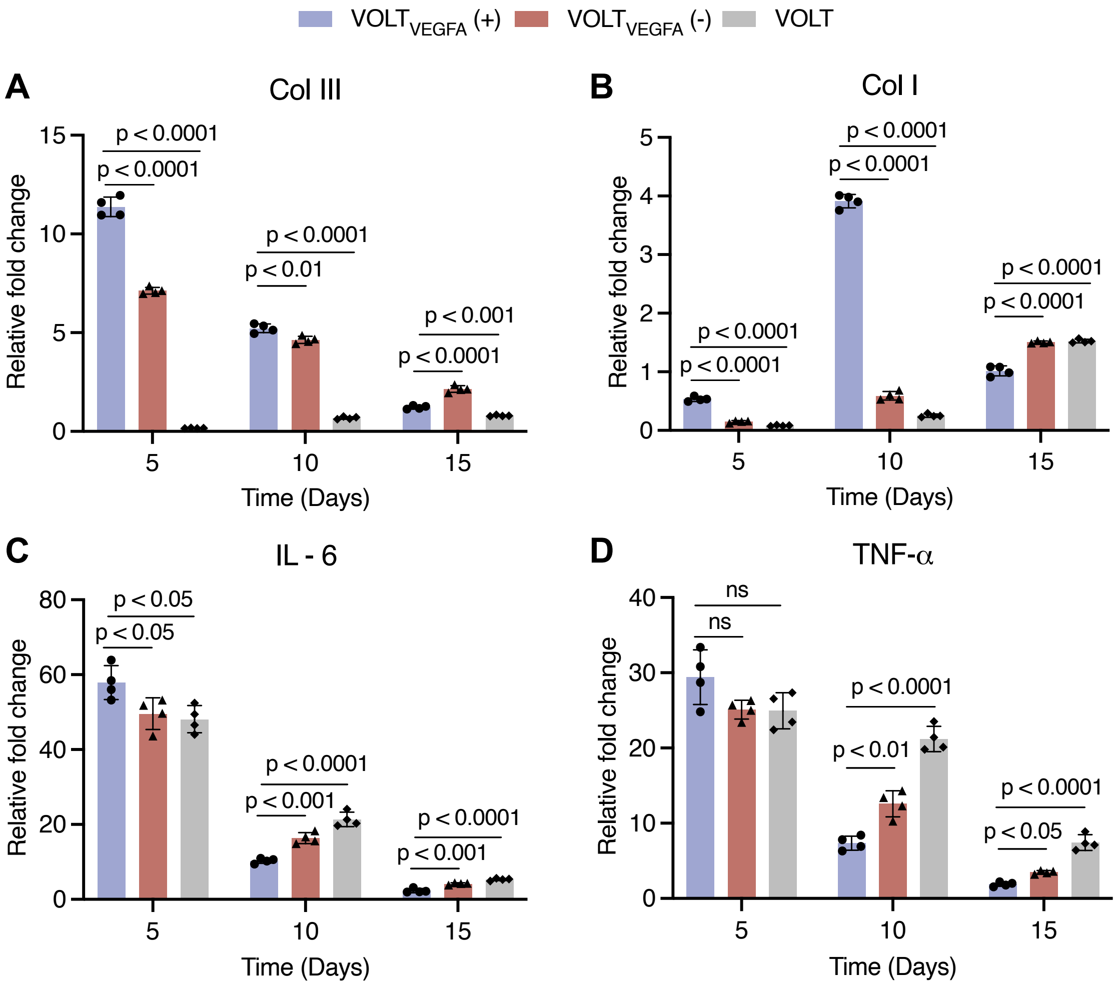

Figure S14 | (A-D) RT-PCR profiling of Col III, Col I, IL-6 and TNF-α gene expression in excised tissue samples collected from the healing areas at pre-defined time points. Data are presented as mean ± SD of n = 4 biologically independent samples. The statistical significance of differences (*p value*) was calculated using one-way ANOVA with Dunnett's multiple comparisons test.

Table S1. Plasmids used and designed in this study

Plasmid	Description	Reference
BB6-BlastR	SB100X-specific transposon containing a constitutive BlastR and iRFP expression unit. (ITR-MCS-pA:P_{hCMV}-BlastR-P2A-iRFP-pA-ITR).	Huang et al.[1]
BB6-PuroR	SB100X-specific transposon containing a constitutive ECFP and PuroR expression unit. (ITR-MCS-pA:P_{RPBSA}-ECFP-P2A-PuroR-pA-ITR).	Huang et al.[1]
BB6-ZeoR	SB100X-specific transposon containing a constitutive ZeoR and mRuby expression unit. (ITR-MCS-pA:P_{hCMV}-ZeoR-P2A-mRuby-pA-ITR).	Huang et al.[1]
pcDNA3.1(+)	Constitutive mammalian expression vector containing a NeoR resistance gene (P_{hCMV}-MCS-pA).	Thermo Fisher Scientific, CA
pCMV-T7-SB100	Constitutive SB100X expression vector (P_{hCMV}-SB100X-pA) (Addgene no. 34879).	Mates et al. [2, 3]
pJH3	Constitutive mammalian SEAP expression vector (P_{hCMV}-SEAP-pA).	Huang et al.[1]
pJH42	Constitutive SB100X expression vector in pcDNA3.1(+) backbone. (P_{hCMV}-SB100X-pA).	Huang et al.[1]
pKEAP1	Constitutive KEAP1 expression vector (PhCMV-KEAP1-3xFlag-pA) (Addgene no. 28023).	Fan et al.[4]
pVEGFA	VEGFA (*Homo sapiens*) in pLenti6.3/V5-DEST	Center for Personalized Diagnostics (GenBank Accession No.

		NM_001025366)
pNRF2	Constitutive NRF2 expression vector (PhCMV-Flag-NRF2-pA) (Addgene no. 36971).	Camp et al.[5]
pJH1003	Constitutive NRF2 expression vector (PhCMV-NRF2-pA).	Huang et al.[1]
pJH1004	Constitutive KEAP1 expression vector (PhCMV-KEAP1-pA).	Huang et al.[1]
pJH1005	ARE-driven SEAP expression vector (P_{DART}-SEAP-pA).	Huang et al.[1]
pJH1054	SB100X-specific transposon containing a constitutive KEAP1 and BlastR expression unit (ITR-PhCMV-KEAP1-P2A-BlastR-pA-ITR).	Huang et al.[1]
pJH1101	SB100X-specific transposon containing a constitutive NRF2 expression unit and a constitutive ECFP and PuroR expression unit (ITR-PhCMV-NRF2-pA:PRPBSA-ECFP-P2A-PuroR-pA-ITR).	Huang et al.[1]
pJH1169	SB100X-specific transposon containing a four tandem ARE-driven SEAP and insulin expression unit and a constitutive ZeoR expression unit (ITR-PDART4-SEAP-P2A-mINS:PmPGK-ZeoR-pA-ITR).	Huang et al.[1]
pPGR30	ARE-driven SEAP and VEGF-A expression vector. (P_{DART}-SEAP- P2A-VEGFA-pA) The target fragment for SEAP was PCR-amplified from pJH1005 with OPGR30_GF-SEAP (5'-CTGTTCGAAGCGGAATTCACCATGACTAGTCTGCTGCTGCTGCTG-3') and OPGR30_GR-p2A-SEAP (5'-CGTCGCCTGCCTGCTTCAGCAGGGAAAAGTTGGTTGCTCCGGTCTGCTCGAATCTGCCGG-3'). Another target fragment for VEGF-A was PCR-amplified from VEGFA plasmid (DNASU Clone no. HsCD00870753) using (5'-	This work

	GCTGAAGCAGGCAGGCGACGTGGAGGAGAATCCTGGACCCATGACGGACAGACAGACAGAC-3’) and (5’-GGCCTCAAAGCTTTCTAGACACCGCTACCGCCTCGGCTTGTCACATCT-3’). The fragments were cloned into pJH1005 (digested by *EcoRI*/ *Hin*dIII) by Gibson assembly.	
pPGR34	SB100X-specific transposon containing a four tandem ARE-driven SEAP and VEGF-A expression unit and a constitutive ZeoR expression unit.<br>(ITR-P_{DART4}-*SEAP*-P2A-*VEGFA*:PmPGK-*ZeoR*-pA-ITR)<br>The target fragment for SEAP was PCR-amplified from pPGR 30 with OPGR30_GF-SEAP (5’-CTGTTCGAAGCGGAATTCACCATGACTAGTCT GCTGCTGCTGCTG-3’) and OPGR30_GR-p2A-SEAP (5’-CGTCGCCTGCCTGCTTCAGCAGGGAAAAGTTGGTTGCTCCGGTCTGCTCGAATCTGCCGG-3’). The second target fragment for VEGF-A was PCR-amplified from pPGR30 using (5’-GCTGAAGCAGGCAGGCGACGTGGAGGAGAATCCTGGACCCATGACGGACAGACAGACAGAC-3’) and (5’-GGCCTCAAAGCTTTCTAGACACCGCTACCGCCTCGGCTTGTCACATCT-3’). The fragments were cloned into pJH1169 (digested by *EcoRI*/ *Hin*dIII) by Gibson assembly.	This work
pPGR35	SB100X-specific transposon containing a four tandem ARE-driven SEAP and VEGF-A expression unit and a constitutive ZeoR and mRuby expression unit.<br>(ITR-P_{DART4}-*SEAP*-P2A-*VEGFA*: P_{hCMV}-ZeoR-P2A-mRuby-pA-ITR)<br>pPGR34 was digested with *Mlu*I/ *Hin*dIII and cloned into BB6-ZeoR (digested by *Mlu*I/ *Hin*dIII) by T4 ligation.	This work

Abbreviations: **VEGF-A:** vascular endothelial growth factor A; **ARE:** antioxidant response element; **BlastR**, gene conferring blasticidin resistance; **CMV**, cytomegalovirus; **ECFP**, enhanced cyan fluorescent protein; **EGFP**, enhanced green fluorescent protein; **iRFP**, near-infrared fluorescent protein; **ITR**, inverted terminal repeats of SB100X; **MCS**, multiple cloning site; KEAP1: Kelchlike ECH-associated protein 1; NRF2: nuclear factor erythroid 2 p45-related factor 2; **mRuby**, a bright monomeric red fluorescent protein; **P2A**, picornavirus-derived ribosome skipping sequence optimized for bicistronic expression in mammalian cells; **pA**, polyadenylation signal; **PCR**, polymerase chain reaction; **P_{CRE},** CRE-containing synthetic mammalian promoter; **P_{hCMV}**, human cytomegalovirus immediate early promoter; **$P_{hCMVmin}$,** minimal version of P_{hCMV}; **P_{RPBSA}**: a constitutive synthetic mammalian promoter; **P_{SV40},** simian virus 40 promoter; **P_{DART},** promoter of DC-actuated regulation technology containing ARE element, O_{ARE}-$P_{hCMVmin}$; **P_{DART4}**: O_{ARE4}-$P_{hCMVmin}$; **PuroR**, gene conferring puromycin resistance; **SB100X**, optimized Sleeping Beauty transposase; **SEAP**, human placental secreted alkaline phosphatase; **ZeoR**, gene conferring zeocin resistance.

Table S2. Primers used for RT-PCR studies

Genes	Forward primer	Reverse Primer
TGF-β	5′ – AGGGCTACCATGCCAACTTC – 3′	5′ – CCACGTAGTAGACGATGGGC – 3′
Col III	5′ – AGAGGCTTTGATGGACGCAA – 3′	5′ – GGTCCAACCTCACCCTTAGC – 3′
Col I	5′ – GGGGCAAGACAGTCATCGAA – 3′	5′ – GGTGGGAGGGAACCAGATTG – 3′
EGF	5′ – GGTCCACCCATTGGCAAAAC – 3′	5′ – CACGAATCCTTCCCGACACA – 3′
FGF	5′ – CAAAACCTGACCCGATCCCT – 3′	5′ – CCGTGACGCAGCTCCTAAA – 3′
TNF-α	5′ – AGGACACCATGAGCACGGAA – 3′	5′ – GGGCCATGGAACTGATGAGA – 3′
IL-6	5′ – AGACTTCCAGCCAGTTGCCT – 3′	5′ – CTGACAGTGCATCATCGCTG – 3′
GAPDH_sense	5′ – TCTCTGCTCCTCCCTGTTCT – 3′	5′ – CTTGCCGTGGGTAGAGTCAT – 3′

Supplementary References

[1] J. Huang, S. Xue, P. Buchmann, A. P. Teixeira, M. Fussenegger, *Nat. Metab.* 2023, 5 (8), 1395.

[2] L. Mátés, M. K. Chuah, E. Belay, B. Jerchow, N. Manoj, A. Acosta-Sanchez, D. P. Grzela, A. Schmitt, K. Becker, J. Matrai, *Nat. Genet.* 2009, 41 (6), 753

[3] E. Kowarz, D. Löscher, R. Marschalek, *Biotechnol. J.* 2015, 10 (4), 647.

[4] W. Fan, Z. Tang, D. Chen, D. Moughon, X. Ding, S. Chen, M. Zhu, Q. Zhong, Autophagy 2010, 6 (5), 614.

[5] N. D. Camp, R. G. James, D. W. Dawson, F. Yan, J. M. Davison, S. A. Houck, X. Tang, N. Zheng, M. B. Major, R. T. Moon, *J. Biol. Chem.* 2012, 287 (9), 6539.
